# Multiplexed Integrin Detection and Cancer Cell Classification Using Multicolor Gap-Enhanced Gold Nanorods and Machine Learning Algorithm

**DOI:** 10.3390/nano15221693

**Published:** 2025-11-08

**Authors:** Suprava Shah, Reed Youngerman, Alberto Luis Rodriguez-Nieves, Mitchell Lee Taylor, William Rodney Bantom, David Thompson, Jingyi Chen, Yongmei Wang, Xiaohua Huang

**Affiliations:** 1 Department of Chemistry, The University of Memphis, Memphis, TN 38152, USA; sshah12@memphis.edu (S.S.); ryngrman@memphis.edu (R.Y.); mltylor3@memphis.edu (M.L.T.); wrbantom@memphis.edu (W.R.B.III); ywang@memphis.edu (Y.W.); 2 Department of Chemistry and Biochemistry, The University of Arkansas, Fayetteville, AR 72701, USA; dt034@uark.edu (D.T.); chenj@uark.edu (J.C.)

**Keywords:** integrin, multiplexed detection, breast cancer, SERS, gap-enhanced gold nanorod, machine learning

## Abstract

Integrins, cell-surface adhesion receptors involved in tumor progression, invasion, and metastasis, serve as crucial biomarkers for cancer diagnosis and therapeutic targeting. Multiplexed detection of integrins and cancer cell classification at the single-cell level allows for comprehensive profiling, facilitating precise identification and categorization of tumor cells that are heterogeneous in integrin expression and cell subtype. In this study, we developed a five-plex detection platform and demonstrated integrin profile for cancer cell classification leveraging surface-enhanced Raman scattering (SERS) with gap-enhanced gold nanorods (GENRs) in conjunction with advanced computational analysis. Specifically, we synthesized GENRs bearing five distinct Raman nanotags, each producing a unique spectral fingerprint upon targeting a specific integrin subtype expressed on cancer cell surfaces. SERS signals from single cancer cells—after labeling simultaneously with the five-color SERS nanotags—were collected on single cells and subsequently analyzed with classical least squares regression to reliably deconvolute and quantify expression level of five different integrin monomers. Utilizing a random forest classifier trained on integrin profiles from individual cancer cell lines, we achieved simultaneous detections of three different breast cancer cell lines, with exceptional classification accuracy of 99.9%. The feasibility of this method for multiplexed detection of circulating tumor cells was tested using peripheral blood mononuclear cells (PBMCs) spiked with mixed breast cancer cells from three cell lines. By integrating GENRs, multiplexed SERS nanotag technology, and machine learning, our platform significantly advances cancer diagnostics through accurate integrin-based cell profiling and classification. These findings highlight the potential of multiplexed integrin detection using SERS technology as a powerful diagnostic approach, ultimately supporting improved cancer subtype characterization, personalized diagnostics, and more targeted therapeutic strategies.

## 1. Introduction

Integrins have been found to play important roles at various stages of cancer progression and metastasis. For instance, integrin-dependent processes are implicated in cancer initiation and proliferation, survivability of circulating tumor cells (CTCs), local and vascular invasion, metastatic colonization of new tissue, and extravasation into secondary site [1]. Integrins drive most metastatic cascades of solid tumors by upregulating the expression of matrix metalloproteinase genes and facilitating protease activation and function at the extracellular matrix (ECM) interface [2,3,4]. Additionally, integrin-linked mechanisms have been found to contribute to the activity of cancer-associated fibroblasts (CAFs), which promote cancer progression by forming pro-migratory tracks within the stromal ECM, ultimately leading to invasion [1,5]. This is particularly relevant in CTCs, which constantly enter and migrate through the bloodstream and lymphatic system after detaching from the primary tumor.

Integrins are type I transmembrane glycoproteins composed of 18 α and 8 β subunits. These transmembrane heterodimers combine to generate 24 functional heterodimers that bind various ligands in the extracellular matrix (ECM) [6,7,8,9]. Extensive studies have shown that integrins are implicated in various aspects of cancer due to their contributions to tumor progression, metastasis, and interaction with the tumor microenvironment [10,11,12,13]. In breast cancer, for example, integrin α3β1 is involved in cell adhesion, migration, and invasion, promoting tumor growth and metastatic dissemination [14]. Integrin β1 is broadly expressed in breast cancer and contributes significantly to cell survival, proliferation, migration, and resistance to chemotherapy [15,16]. Integrin β4 is prominently associated with aggressive breast cancer subtypes, notably triple-negative breast cancer, and enhances tumor invasion, migration, and resistance to apoptosis [17,18].

Thus, integrin detection and characterization are vital for cancer detection and understanding of the molecular mechanisms underlying cancer progression, metastasis, and therapeutic resistance. Particularly, multiplexed detection of integrins at the single-cell level is essential for accurately characterizing the heterogeneous expression patterns that exist within tumor populations. Different integrins play distinct roles in regulating cancer cell behavior, including adhesion, migration, invasion, and response to therapy. Since individual tumor cells may express unique combinations of integrins, single-cell multiplexed analysis enables precise profiling that bulk measurements cannot achieve.

Fluorescence-based multiplexed single-cell molecular detection has advanced significantly, enabling simultaneous profiling of many different targets within individual cells [19,20]. Innovations such as spectral unmixing, cyclic staining, and DNA-barcoded antibodies have greatly expanded multiplexing capacity beyond traditional fluorophore limitations, thereby facilitating insights into cellular heterogeneity, tissue architecture, and disease mechanisms [21,22,23,24,25,26,27]. Due to their unique attributes, surface-enhanced Raman scattering (SERS) nanotags—plasmonic nanoparticles coated with Raman reporters—have emerged as a powerful new class of optical labels for multiplexed detection of molecular targets [28]. SERS provides exceptionally high sensitivity, enabling detection of analytes at ultra-low concentrations, down to single-molecule levels, due to strong electromagnetic enhancement near plasmonic nanostructures [29,30,31]. SERS produces narrow, distinct spectral fingerprints, allowing for simultaneous detection and clear discrimination of multiple analytes or biomarkers without significant spectral overlap [32,33,34,35,36,37,38,39,40,41]. Moreover, SERS-based assays offer exceptional photostability, resistance to photobleaching, and compatibility with a wide range of biological environments, thereby serving as a powerful and noninvasive platform for biomedical detection and analysis [42,43,44]. These properties collectively make SERS a powerful technique for accurate, sensitive, and reliable multiplexed biomarker detection applications, particularly valuable for cancer diagnostics and profiling at the single-cell level. However, a major limitation of traditional SERS nanotags is the desorption or diffusion of surface-adsorbed Raman reporters over time, leading to signal instability and reduced detection reliability.

Unlike traditional SERS nanotags, gap-enhanced Raman tags (GERTs) protect Raman reporters from diffusion by a thin layer of metallic shell that also helps enhance the Raman signals of the reporters due to strong electromagnetic field confinement within the nanogap between the core and the shell [45]. Owing to these advantages, growing interest has been devoted to the synthesis, characterization, and biomedical applications of GERTs with diverse morphologies such as spheres, cucumbers, dumbbells, petal-like, and spiked rods [46,47,48,49,50,51,52,53,54,55]. Rod-shaped core–shell nanostructures are particularly intriguing because they generate strong localized electromagnetic fields arising from their anisotropic shape, which enables tunable plasmonic resonance and stronger Raman signal amplification [56]. Accordingly, previous studies have fabricated rod-in-shell particles using AuNRs as the core and silver (Ag) as the shell [57,58,59,60,61,62,63,64].

In this work, we report for the first time the synthesis and application of gap-enhanced AuNRs (GENRs) and their applications for multiplexed integrin detection and cancer cell classification, assisted by machine learning analysis. Using GENRs encoded with different Raman tags, we demonstrate simultaneous detection of five different integrins on single cells. Using a ML model trained with integrin profiles from individual breast cancer cell lines, we achieved simultaneous identification of three breast cancer subtypes spiked into peripheral blood mononuclear cells (PBMCs). By integrating GENRs, multiplexed SERS nanotag technology, and ML algorithms, our platform significantly advances cancer diagnostics through highly accurate integrin-based single-cell profiling and classification. These findings highlight the potential of composite integrin markers as a reliable diagnostic approach, ultimately supporting better cancer subtype characterization, personalized diagnostics, and more targeted therapeutic strategies.

## 2. Materials and Methods

### 2.1. Materials

Unless otherwise specified, all chemicals were purchased from Millipore-Sigma (Burlington, MA, USA) including Raman reporters 3,3′-Diethylthiacarbocyanine iodide (DTDC; purity 98%) and silicon 2,3-naphthalocyanine dihydroxide (SiNC. purity 80%). QSY21 (the succinimidyl ester format), cell dissociation buffer, goat anti-mouse IgG conjugated with horseradish peroxidase (HRP), and bovine serum albumin (BSA) were purchased from Fisher Scientific (Waltham, MA, USA). QXL680 was purchased from Anaspec (Fremont, CA, USA). BHQ3-amine was purchased from Biosearch Technologies (Dexter, MI, USA). Mouse integrin α3 (clone ASC-1), β1 (clone TS2/16), β3 (clone VI-PL2), β4 (clone 58XB4), and β5 (clone AST-3T) anti-human monoclonal antibodies, mouse monoclonal isotype IgG control antibody (clone MG1-45), and density gradient medium Lymphopure™ were purchased from BioLegend (San Diego, CA, USA). Thiolated methoxy polyethylene glycol (mPEG-SH. MW = 5000. Purity: >95%) and thiolated polyethylene glycol N-hydroxy succinimide (NHS-PEG-SH, MW = 1000) were purchased from Nanocs (New York, NY, USA). Fetal bovine serum (FBS) and cancer cell lines SKBR3, MDA-MB-231 (MM231), and MCF7 were purchased from ATCC (Manassas, VA, USA). Dulbecco’s modified Eagle’s medium (DMEM) with high glucose (4.5 g/L), RPMI 1640 medium, 0.25% trypsin, 100 U/mL HyClone penicillin–streptomycin, 1-Step ultra-3,3′,5,5′-tetramethyl-benzidine (TMB)-enzyme-linked immunosorbent assay (ELISA) substrate solution, 4′,6-diamidino-2-phenylindole (DAPI), and centrifugal filter (10 kDa MWCO) were purchased from Avantor (Allentown, PA, USA). Janus Green cell normalization stain was purchased from Abcam (Cambridge, UK).

### 2.2. Synthesis of Gold Nanorods (AuNRs)

AuNRs were synthesized following a method described in our previous work [65]. Specifically, the method involves two steps, preparation of a Au seed solution and growth of AuNRs. To synthesize Au seed nanoparticles, a freshly prepared solution of sodium borohydride (NaBH_4_, 10 mM) was initially chilled at 4 °C for 20 min. Concurrently, a solution of hexadecyltrimethylammonium bromide (CTAB, 0.20 M, 1.5 mL) was prepared under constant stirring at 37 °C until complete dissolution. Subsequently, chloroauric acid (HAuCl_4_, 1.0 mM, 0.50 mL) was added to the CTAB solution, and the mixture was stirred at room temperature (RT) for 5.0 min. Following this period, 120 µL of the cooled NaBH_4_ solution was rapidly injected under vigorous stirring. Stirring continued for an additional 3.0 min until the solution transitioned from golden yellow to brown, signaling the formation of Au seeds approximately 2.0 nm in diameter [66]. The resulting seed solution was subsequently incubated undisturbed at 27 °C for 5.0 h, ensuring complete decomposition of residual NaBH_4_. This reaction facilitates the reduction of Au^3+^ ions to atomic Au^0^, subsequently aggregating into gold nanoparticles.

In a separate vial, a growth solution was prepared by mixing CTAB (0.20 M, 10 mL) with HAuCl_4_ (1.0 mM, 10 mL), which was stirred for 5 min. Afterward, silver nitrate (AgNO_3_, 4.0 mM, 300 µL) was added to the mixture and stirred for an additional 5.0 min. L-ascorbic acid (AA, 79 mM, 140 µL) was then introduced, causing a reduction of Au^3+^ ions to Au^1+^, evident by the solution turning colorless from its initial golden yellow hue. It is critical to avoid complete reduction of Au^1+^ to Au^0^ at this stage in order to prevent secondary nucleation during subsequent growth. After stirring for 2.0 min post-AA addition, 24 µL of the previously synthesized Au seed solution was injected, followed by gentle stirring for 30 s. The solution was finally incubated at 27 °C in a water bath for 5.0 h to facilitate the controlled growth of AuNRs. Post-incubation, the AuNRs were concentrated by centrifugation at 8830× *g* for 10 min ( Centrifuge 5415C, Eppendorf, Hamburg, Germany). The supernatant was discarded, and the AuNR pellets were redispersed in ultrapure water.

### 2.3. Synthesis and Characterization of Multicolor Gap-Enhanced Gold Nanorods (GENRs)

In a 1.5 mL centrifuge vial, 175 µL of AuNRs (2.0 nM) was combined with specified Raman reporters, QSY21 (3.0 µL, 0.10 mM), BHQ3 (5.0 µL, 1.0 mM), DTDC (10 µL, 1 mM), SiNC (40 µL, 0.10 mM), or QXL680 (60 µL, 0.10 mM). The stock solutions of these Raman reporters were prepared using dimethyl sulfoxide (DMSO) for QSY21, DTDC, SiNC, and QXL680 and ultrapure water for BHQ3. The resulting mixtures were sonicated for 30 min to ensure homogeneous and efficient adsorption of the Raman reporters onto the AuNR surface. Reporter to AuNR ratios were carefully optimized to generate comparable SERS intensities at their characteristic Raman shifts: BHQ3 at 1094 cm^−1^, SiNC at 684 cm^−1^, QXL680 at 1140 cm^−1^, DTDC at 510 cm^−1^, and QSY21 at 1496 cm^−1^. Subsequently, the Raman reporter-loaded AuNRs (SERS-AuNRs) were purified via centrifugation at 8160× *g* for 10 min, followed by two washing steps with 0.05 M CTAB solution to eliminate residual dye molecules and prevent nanoparticle aggregation. The purified SERS-AuNR pellets were redispersed in 175 µL of fresh 0.050 M CTAB solution to yield stable SERS-active nanoparticles.

The GENRs were synthesized following the method of Zhang et al. with modifications [50]. Briefly, 16 mL of 0.050 M CTAB solution was combined with 960 µL of 4.0 mM HAuCl_4_ solution and stirred magnetically for 5.0 min. Subsequently, 480 µL of 10 mM AA was added, inducing the reduction of Au^3+^ to Au^1+^, observable by the disappearance of the characteristic yellow color after approximately 2.0 min. Immediately thereafter, 175 µL of the previously prepared SERS-AuNR suspension was introduced, and the solution was stirred briefly for 30 s. The mixture was incubated at 37 °C for 10 min, during which a distinct color change to blue confirmed successful gold encapsulation. The concentrations of HAuCl_4_, AA, and seeds were varied to investigate their impact on the formation of GENRs. The resulting GENRs were purified by centrifugation at 8830× *g* for 10 min and resuspended in 175 µL of ultrapure water pending further characterization and use.

The absorption spectra were measured using a Spectronic 200E UV-Vis spectrometer (Thermo Fisher Scientific, Madison, WI, USA). Particle size and concentration were determined by nanoparticle tracking analysis (NTA) using a NanoSight LM10 microscope (Malvern Instruments) and dynamic light scattering (DLS) with a Zetasizer Nano ZS (Malvern Instruments). The size and morphology of AuNRs and GENRs was examined using a transmission electron microscope (TEM) (JEM-1011, JEOL Ltd., TYO, Japan).

### 2.4. Antibody Conjugation

Prior to conjugation onto GENRs, target-specific monoclonal antibodies (anti-α_3_, anti-β_1_, anti-β_3_, anti-β_4_, and anti-β_5_) were functionalized with thiol groups following an established protocol developed in our previous work [34,35]. Briefly, the anti-α3, anti-β1, anti-β3, anti-β4, or anti-β5 antibodies were first exchanged with bicarbonate buffer by mixing 40 µL, 0.50 mg/mL of antibodies and 160 µL of bicarbonate buffer (0.20 M, pH 9.0). The solution was then added to a centrifugal filter (10 kDa MWCO) and centrifuged at 13,800× *g* (Eppendorf 5415C centrifuge) for 3.0 min. Antibodies were resuspended in 40 µL of bicarbonate buffer and incubated with NHS-PEG-SH 1000 (2.7 µL, 5 mM in DPBS) at 37 °C for 2.0 h. The reaction was quenched by adding 100 µL of Tris buffer (pH 8.0). The resulting thiolated proteins were washed three times by filtration with a centrifugal filter (10 kDa MWCO) at 13,800× *g* for 3.0 min and resuspended in 200 µL of HEPES buffer. Finally, the thiolated proteins were resuspended in 20 µL of HEPES buffer and mixed with GENRs (175 μL, 150 pM) for 16 h at 4 °C. After incubation, the mPEG-SH 5000 (7.9 µL, 100 µM) was introduced and incubated with constant stirring on a vortex mixer for 2.0 h at RT. The resulting conjugates were purified by three rounds of centrifugation at 8160× *g* for 10 min and washing with DPBS containing 0.050% Tween-20 (DPBST). Finally, the purified conjugates were redispersed in 175 μL of DPBST containing 0.050% sodium azide and stored at 4 °C until further use.

### 2.5. Cell Culture and Collection

SKBR3, MM231, and MCF7 breast cancer cell lines were cultured in their respective media (RPMI-1640 for SKBR3, DMEM for MM231 & MCF7) with 10% FBS, 100 U/mL penicillin/streptomycin, 1% sodium pyruvate, at 37 °C with 5% CO_2_. Cells were cultured in a 6-well culture plate at a density of 5 × 10^4^ cells/well and incubated for 48–72 h. The cells were then washed with DPBS and incubated in cell dissociation buffer for 10 min at 37 °C. The dislodged cells were centrifuged at 390× *g* ( IEC Centra CL2 centrifuge, ThermoFisher Scientific, Waltham, MA, USA) for 5 min and resuspended in DPBS for immediate use. Cells were counted using a Hausser Scientific™ Bright-Line™/Hy-Lite™ Counting Chamber hemocytometer.

### 2.6. Characterization of Integrin Expression with Cellular ELISA

In a typical assay, approximately 50 μL medium containing 2000 cells were added into a well of the 96-well ELISA plate and incubated for 24–48 h at 37 °C with 5% CO_2_. After washing with DPBST, the cells were fixed in 50 μL of 4.0% paraformaldehyde for 15 min at 37 °C. The cells were washed with DPBS and blocked with 200 μL of 5.0% BSA for 1.0 h at 37 °C. After washing with DPBST, 50 μL of 2.0 μg/mL primary antibody or isotype IgG was added and incubated for 1.0 h at 37 °C. After washing three times with DPBST and two times with DPBS, 50 μL of 1:60 goat anti-mouse HRP-conjugated secondary antibody was added and incubated for 1.0 h at 37 °C. Following the same washing method, 100 μL of TMB was added and incubated for 15 min at 37 °C. The reaction was stopped by adding 100 μL of 2.0 M sulfuric acid. The absorbance was measured at 450 nm using a microplate reader (ELx800, BioTek Instruments, Inc., Winooski, VT, USA). Following absorbance measurement, the cells were washed three times with DPBS, and 50 μL of 1× Janus green dye was added and incubated for 5.0 min at RT. After washing with DPBS five times, 100 μL of 0.50 M HCl was added and incubated for 10 min at RT. The absorbance was then measured at 595 nm. Each experiment was performed in triplicate, and the mean value with standard deviation was reported in all studies.

### 2.7. Collection of PBMCs from Human Whole Blood

Human whole blood from healthy female donors was purchased from BioIVT (Hicksville, NY, USA). To collect PBMCs from whole blood, 5.0 mL of blood was first diluted with 5.0 mL of EasyStep Buffer (StemCell Technologies, WA, USA) in a 1:1 ratio. The diluted blood was then added to 5.0 mL of Lymphopure™ density gradient medium and the sample was centrifuged for 20 min at 800× *g* (5804R centrifuge, Eppendorf, Hamburg, Germany). After centrifugation, the plasma was carefully removed and the PBMC layer located between the plasma and density gradient buffer was collected and transferred to a 1.5 mL protein Lo-Bind centrifuge tube for immediate use.

### 2.8. Isolation and Characterization of Spiked Cancer Cells in PBMCs

100 µL PBMCs containing 5.0 × 10^4^ breast cancer cells (SKBR3, MM231, or MCF7) was fixed in 1.0% paraformaldehyde for 15 min at RT. The sample was centrifuged at 390× *g* for 5 min and the cell pellet was resuspended in 100 μL of DPBS. To deplete white blood cells (WBCs) using magnetic nanobeads, 10 μL of MojoSort™ Human CD45-conjugated Nanobeads (BioLegend) was added and incubated for 15 min on ice, followed by magnetic separation using a Qiagen 12-tube magnet for 10 min. The supernatant containing the cancer cells was collected and stored at 4 °C for further use. To simultaneously detect cancer cells from different cell lines in PBMCs, 100 μL PBMCs was spiked with SKBR3, MM231, and MCF7 cells, each 5.0 × 10^4^ cells. WBCs were depleted from the spiked PBMCs following the procedure described above.

To characterize the efficiency of WBC depletion, the solution was transferred to a centrifuge vial and incubated with 300 nM DAPI for 5.0 min at RT followed by purification via centrifugation (390× *g*, 5.0 min). The cells were resuspended in 500 μL of DPBS containing 1.0 μg/mL fluorescein isothiocyanate (FITC)-conjugated CD45 antibodies and incubated for 30 min at RT. The cells were purified again via centrifugation and resuspended in 100 μL of DPBS. Cells were examined with an Olympus IX 71 microscope with the DAPI filter (excitation wavelength = 387/11 nm; emission wavelength = 447/60 nm) and FITC filter (excitation wavelength = 482/35 nm; emission wavelength = 536/40 nm) to recognize nucleated cells and WBCs, respectively. The fluorescence images were analyzed with auto-SEDIA, a method we developed recently for mask-target dual imaging analysis [67]. The DAPI image was used as the mask, and the FITC image was used as the target to count the number of WBCs in the DAPI image.

### 2.9. Cell Labeling and Signal Collection

To examine the cellular binding of each antibody-GENR conjugate and the IgG control nanoparticles, cells (SKBR3, MM231, or MCF7) were cultured in an 8-well Nunc™ Lab-Tek™ II Chamber Slide (ThermoFisher Scientific, Waltham, MA, USA). After overnight incubation, cells were washed with DPBST and fixed with 150 µL of 1% paraformaldehyde for 15 min. The fixed cells were washed with DPBS and were blocked with 200 µL of 1% BSA for 30 min at 37 °C. The cells were washed again with DPBST followed by incubation with 150 µL of fresh medium containing 20 pM specified conjugates at 37 °C for 1 h. Finally, the cells were washed sequentially with DPBST and DPBS.

To label the cancer cells for each cell line with GENRs for Raman characterization, 100 μL of DPBS containing 5.0 × 10^4^ cells (SKBR3, MM231, or MCF7) was mixed with the five-color antibody-conjugated GENRs (13 pM final concentration for each conjugate) for 30 min at 37 °C with gentle mixing on a vortex mixer. The cells were then fixed with 1.0% paraformaldehyde for 5.0 min and purified by low-speed centrifugation (390× *g*, 5.0 min), during which only cells were pelleted. The resulting cell pellet was resuspended in 100 µL of DPBS and used for Raman measurements.

To label the spiked cancer cells for each cell line in PBMCs, 100 μL of WBC-depleted PBMCs containing 5.0 × 10^4^ cells (SKBR3, MM231, or MCF7) was mixed with 500 μL of DPBS containing 300 nM DAPI at RT for 5.0 min. Following purification via centrifugation, the cells were resuspended in 500 μL of DPBS containing 1 μg/mL FITC-conjugated CD45 antibodies and incubated for 30 min at RT. The cells were purified again via centrifugation and resuspended in 100 μL of DPBS containing the five-color antibody-conjugated GENRs (13 pM for each conjugate) for 30 min at 37 °C with gentle mixing. Following the reaction, the cells were fixed with 1.0% paraformaldehyde, purified by centrifugation, and resuspended in 100 μL of DPBS for characterization. To label the mixed cancer cells in spiked PBMCs, 100 μL of WBC-depleted PBMCs containing SKBR3, MM231, and MCF7 cells (each 5.0 × 10^4^ cells) was labeled with DAPI, FITC-conjugated CD45 antibodies and the antibody-conjugated GENRs following the same procedures as described above for labeling the cancer cells for the individual cell lines.

Fluorescence images and SERS signals were collected using an assembled dual fluorescence/Raman microscope based on an CX41 fluorescence microscope (Olympus American Inc., Center Valley, PA, USA) and a portable Raman spectrometer ( ProRaman L spectrometer, TSI Inc., Shoreview, MN, USA) with laser excitation at 785 nm coupled with a CCD detector. Fluorescence images were collected with a DAPI filter to visualize cell nuclei and an FITC filter to visualize WBCs. SERS signals were only collected on the cells that were both DAPI-positive and CD45-negative. Single-cell SERS spectra were acquired using a 40× objective with a laser spot size of ~30 µm, sufficient to encompass an entire cell and thus enabling signal collection from each cell in a single spectrum. The laser power was 50 mW, and the acquisition time was 10 s. Spectra were recorded over the 250–3000 cm^−1^ range at a resolution of 7 cm^−1^. Baseline correction was performed automatically by the acquisition software (Pro Raman Reader v8.2.9) using multi-segment polynomial fitting to subtract the broad continuum SERS background. The CCD detector was cooled to −60 °C to minimize thermal noise, and a boxcar smoothing filter was applied for spectral denoising. The processed Raman spectra were displayed as intensity versus Raman shift (cm^−1^). For experiments with individual cell lines, spectra from 300 cells (collected in triplicate experiments) were recorded, while 450 cells were analyzed for mixed cancer cell samples.

### 2.10. Signal Deconvolution and Data Analysis

SERS signals from single cells were deconvolved using the SOLO + MIA 9.5 software (Eigenvector Research Inc., Manson, WA, USA) by direct classical least-squares regression (CLS) to obtain signals from each Raman tag. The S_total_ obtained from a mixture represents a linear addition from the individual nanotags S_1_–S_5_. Specifically, S_total_ = C_1_S_1_ + C_2_S_2_ + C_3_S_3_ + C_4_S_4_ + C_5_S_5_ + Δ, where C_1_–C_5_ is the weight factor for the individual nanotags, and Δ is the minimized residual error for optimal weight factor choices. The weight factors can be interpreted as a relative SERS signal intensity to the reference SERS spectrum of the pure nanotag solution. This reference spectrum was collected using 5 μL of 20 pM for each colored Raman tag with a 100 μm Raman probe. The laser power was 25 mW, and the acquisition time was 1 s.

### 2.11. Machine Learning, Statistics, and Clustering Analysis

The t-distributed stochastic neighbor embedding (t-SNE) technique from Python’s scikit-learn library 1.7.2 was employed to visualize the clustering of cells based on deconvoluted multicolor SERS data. Data from all five cell lines were compiled into a single Pandas DataFrame, where each deconvoluted Raman signal was assigned to one of five columns. A total of 60 t-SNE mappings were generated at varying perplexity values ranging from 5 to 300. For each perplexity value, five mappings were created, and the most representative plot was selected based on the lowest Kullback–Leibler divergence value. To determine the final t-SNE mapping and the optimal perplexity value, we identified the plot with the lowest Davies–Bouldin index (DBI), ensuring the best representation of the data.

For classification, a random forest model was implemented using the Random Forest Classifier from the scikit-learn library. The dataset, which was uniformly distributed across all cell lines, was split into 70% training data and 30% test data. The model underwent 200 training iterations using default values for both the maximum depth and number of estimators. To evaluate model performance, the average F1 score, and confusion matrices were used as key metrics. After training, the model was retrained on the full training dataset, and this optimized model was subsequently used to predict the class of each cell within a multiplex detection scheme. To visually assess the model’s effectiveness, a t-SNE plot was generated, while a linear correlation plot was created to illustrate the relationship between the mean values of the deconvoluted predicted cell data and the corresponding values from the training dataset.

## 3. Results and Discussions

### 3.1. Overview of the Approach

The ability to simultaneously detect and classify different subtypes of cancer cells is crucial for accurately capturing tumor heterogeneity, thereby enabling precise cancer diagnosis, personalized treatment decisions, and improved patient outcomes. In this study, we explore the feasibility of a multiplexed platform based on cell-surface integrin profiling to detect and differentiate three subtypes of mimic CTCs using breast cancer as a representative the disease model.

**Figure 1:**
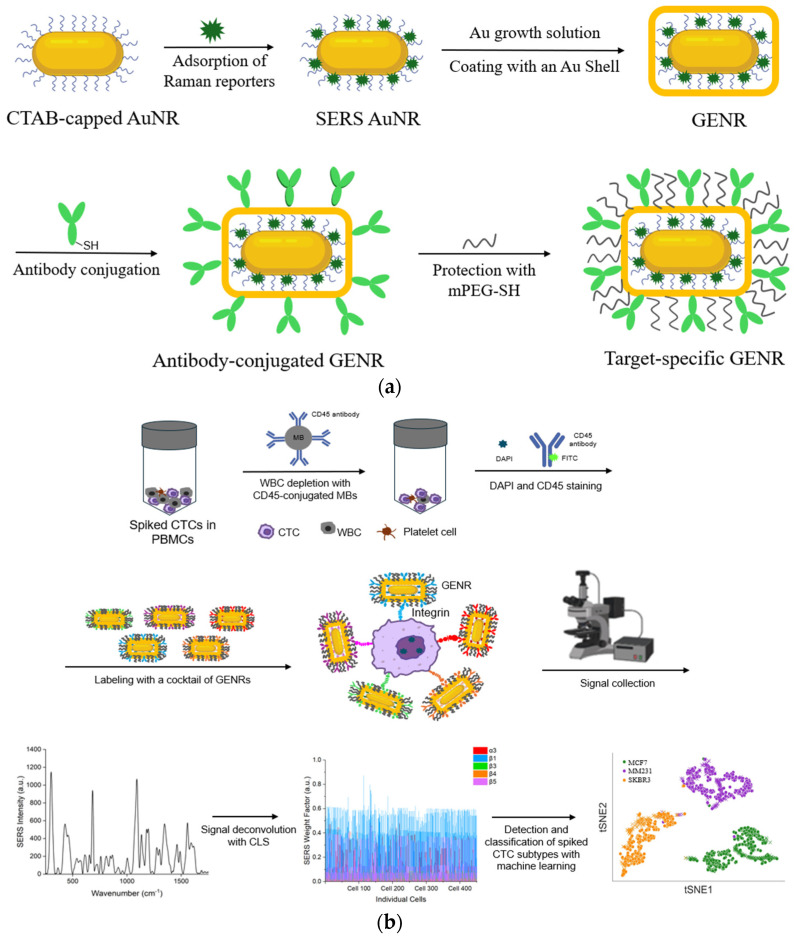
Schematic of multiplexed integrin detection and cancer cell classification using multicolor GENRs and machine learning algorithm. (**a**) Synthesis and antibody conjugation of GENRs to detect a target-specific integrin (α3, β1, β3, β4, or β5) with distinct Raman reporters (SiNC, BHQ3, QXL680, QSY21, and DTDC, respectively). GENRs were synthesized via a two-step procedure, in which Raman reporters were first adsorbed onto CTAB-capped AuNRs and subsequently encapsulated by an external gold shell through a seed-mediated growth method. They were linked with target-specific antibodies via a thiolated PEG linker and saturated with mPEG-SH 5000. (**b**) Simultaneous detection of 5 integrin markers (α_3_, β_1_, β_3_, β_4_, and β_5_) and three breast cancer subtypes (MCF7, MM231, and SKBR3) using the 5 color GENRs in conjunction with machine learning—based data analysis. PBMCs spiked with breast cancer cells (single or mixed cell lines) were subjected to WBC depletion using CD45-conjugated magnetic beads. Following nuclear (DAPI) and WBC (FITC-CD45) staining, CTCs were labeled with five-color GENRs, purified by centrifugation, and analyzed by dual fluorescence-Raman microscopy. Single-cell SERS spectra were deconvoluted using classical least squares (CLS) regression and subsequently classified via machine learning to identify distinct cell lines.

Figure 1 shows an overview of the approach. To recognize cell-surface integrins, we synthesized GENRs in which Raman reporters were incorporated in the gap between the AuNR core and its external Au layer. We utilized GENRs instead of traditional SERS nanotags bearing surface-coated Raman reporters as the unique core–shell architecture of GENRs provides significantly stronger and more stable SERS signals, thereby enhancing detection sensitivity and reliability. Using five different Raman reporters (BHQ3, QSY21 QXL680, DTDC, and SiNC), GENRs with five distinct spectral colors were generated, each reporting a specific integrin marker through conjugation with monoclonal antibodies.

PBMCs is the middle layer of a blood sample following density gradient centrifugation, accounting for 1% or less of the total blood volume. CTCs and WBCs have densities 3.5 to 4.5 times lower than those of red blood cells. Thus, CTCs reside in the PBMC layer after centrifugation of whole blood. CTCs in PBMCs are over ten times more concentrated than in whole blood, which facilitates the detection of rare cells. To mimic patient-derived CTCs, we spiked breast cancer cells into PBMC samples to demonstrate the feasibility of our approach. To test the ability of our method to simultaneously detect cancer cells across multiple subpopulations, we spiked breast cancer cells from three different cell lines into the PBMCs. Prior to integrin labeling, WBCs were depleted using magnetic beads conjugated with anti-CD45 antibodies, which selectively bind to the pan-leukocyte marker CD45, thereby enabling efficient removal of WBCs.

The WBC-depleted cancer cells were then labeled with antibody-GENRs to detect surface integrin markers via SERS signals (Figure 1b). Due to their strong association with breast cancer, five integrins (α3, β1, β3, β4, and β5) were chosen for this study. Each of these integrins was reported by a distinct GENR color through its incorporated Raman tag. SERS signals collected from single cells were a combination of the signals from the five-color GENRs. The mixed signals were deconvoluted using CLS regression to resolve the signals contributed by each integrin marker. This enabled simultaneous detection of five different integrin markers on the same cell. To ensure accuracy in detection, negative staining with DAPI and FITC-CD45 was applied before GENR labeling to identify nucleated cells and WBCs, respectively. Only the nucleated and CD45-negative cells, i.e., cancer cells, were subjected to SERS measurement.

To deconvolute different cancer cell types within a mixture, a random forest model was implemented using the RandomForestClassifier from the scikit-learn library. The model was trained using integrin profiling data from individual cell lines. The model performance was evaluated using the average F1 score and confusion matrices. To visually assess the model’s effectiveness, a t-SNE plot was generated, and a linear correlation plot was also created to illustrate the relationship between the mean values of the deconvoluted predicted cell data and the corresponding values from the training dataset.

### 3.2. Synthesis and Characterization of GENRs

**Figure 2:**
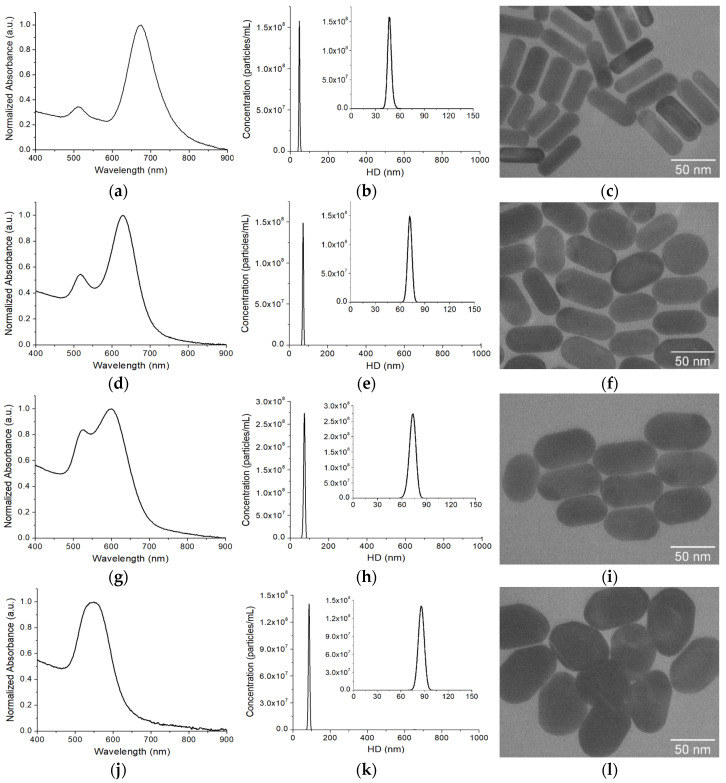

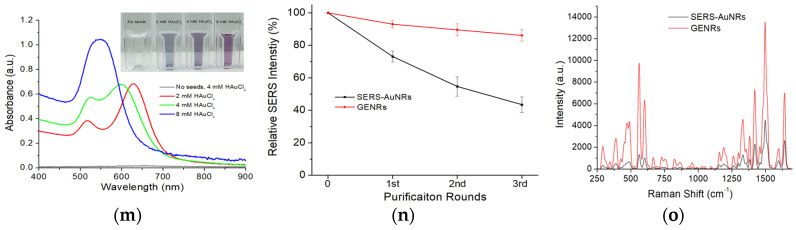
Characterizations of the optical property, size, and morphology of AuNRs and GENRs. Normalized absorption spectrum (**a**,**d**,**g**,**j**), size distribution measured by NTA (**b**,**e**,**h**,**k**), and TEM images (**c**,**f**,**i**,**l**) of AuNRs (**a**–**c**) and GENRs (**d**–**l**) prepared with 2 mM HAuCl_4_ (**d**–**f**), 4 mM HAuCl_4_ (**g**–**i**), and 8 mM HAuCl_4_ (**j**–**l**) in the growth solution. In (**b**,**e**,**h**,**k**), data was represented as the average from three measurements. Inset plot shows the zoomed-in view of the size distribution in the 0–150 nm region. (**m**) Comparison of the unnormalized absorption spectra of as-prepared GENRs under different conditions. (**n**) Comparison of the effects of centrifugation and washing on the SERS signals of SERS-AuNRs and GENRs (prepared with 4 mM HAuCl_4_ in the growth solution). Error bar represents standard deviation from five repeated experiments. (**o**) Comparison of the SERS spectra between PEGylated GENRs (prepared with 4 mM HAuCl_4_ in the growth solution) and SERS-AuNRs at the same nanotag concentration (0.5 nM), which illustrates approximately threefold stronger SERS intensity for GENRs.

GENRs were synthesized following a three-step procedure: (1) synthesis of AuNRs, (2) adsorption of Raman reporters to make SERS-AuNRs, and (3) growth of the SERS-AuNRs in a Au growth solution to form GENRs (Figure 1a). Following the classic seed-mediated growth method, we synthesized AuNRs with a localized surface plasmon resonance (LSPR) band at 676 nm (Figure 2a). The hydrodynamic diameter (HD) of AuNRs, determined by NTA characterization, was 47.0 ± 0.6 nm from triplicate measurements (Figure 2b). The average dimensions of the AuNRs were 53.9 ± 5.7 nm in length and 20.0 ± 3.5 nm in width, based on measurements of over 50 individual AuNRs from multiple TEM images (Figure 2c). The average aspect ratio (AR, length/width) was 2.8 ± 0.6. To make SERS-AuNRs, Raman reporters were adsorbed onto AuNRs via the CTAB capping agent through electrostatic and hydrophobic interactions. After removing free Ramant tags by two rounds of centrifugations and washings with ultrapure water, the SERS-AuNRs were used as seeds to synthesize GENRs using the same method as for AuNRs, except without the addition of AgNO_3_ in the growth solution. The procedure was optimized by investigating the effect of key synthetic parameters on the SERS activity using QSY21 as the model Raman reporter, including the concentrations of Au precursor (HAuCl_4_), reducing agent (AA), and seeds (SERS-AuNRs) in the growth solution.

Figure S1 shows the effect of HAuCl_4_ concentration on the formation of GENRs. Using DLS, we determined that the HD of GENRs increased from ~60 nm to ~80 nm when the HAuCl_4_ concentration increased from 1 mM to 8 mM (Figure S1a). Since the HD of AuNRs was ~42 nm, the shell thickness of the GENRs in terms of hydrodynamic size increased approximately from ~9 nm to ~19 nm. Accordingly, increasing the shell thickness led to a blue shift in the LSPR of GENRs (Figure S1b) and a visible color change in the nanoparticle solution (Figure S1c). Interestingly, increasing the thickness of the Au shell did not cause a continuous uptrend in the SERS activity (Figure S1d). The GENRs prepared with 4 mM HAuCl_4_ showed highest SERS intensity. Similar phenomena were observed previously by Guo et al. in the synthesis of Au-Ag core–shell nanoparticles [68]. In their work, AuNRs with a Ag shell of 12 nm provided the best SERS activity compared to those with thinner or thicker shells. This is not surprising, since the Raman reporters were protected by a hard Au shell. The thickness of the Au shell determines the plasmonic effect of the core–shell structure, the absorption efficiency of the incident Raman laser by the reporters, and the efficiency of Raman scattering light escaping the Au shell for detection. An optimal Au shell exists by a combinatorial effect of these factors.

In contrast to the Au precursor, the reducing agent AA did not cause significant changes on the size and thus the LSPR wavelength of the GENRs (Figure S2a–c). However, increasing AA concentration from 10 mM to 20 mM and higher led to the formation of a greater number of GENRs, as reflected by the increased optical density of LSPR bands at higher AA concentrations. Increasing AA concentration from 10 mM to 40 mM led to a decrease in the SERS intensity (Figure S2d). Further increasing the AA concentration then increased SERS intensity. This could be due to the variation in GENR concentration at different AA levels, as shown in Figure S2b.

Figure S3 shows the results on how seed concentration affects the formation of GENRs. Typically, increasing the concentration of the SERS-AuNR seeds yielded smaller GENRs and thus thinner Au shell (Figure S3a). This is not surprising, as the amount of Au precursor per seed is lower when more seeds are added to the growth solution. As a result of the decrease in the thickness of the Au shell, the LSPR of the GENRs showed a red shift and a color change from purple to blue (Figure S3b,c). The SERS signal decreased when the seed concentration increased from 0.25 nM to 0.50 nM (Figure S3d). However, further increasing the seed concentration increased the SERS intensity again. This is likely due to the combined effects of increased GENR concentration and reduced shell thickness.

Under the optimized conditions (4.0 mM HAuCl_4_, 10 mM AA, and 2.0 nM seeds), we synthesized the GENTs for the multiplexed SERS detection. For comparison, GENRs synthesized under the same optimized conditions, except with 2.0 mM and 8.0 mM HAuCl_4_ in the growth solution, were also synthesized and characterized to evaluate the effect of Au precursor concentration on their optical and structural properties. Figure 2d–l shows the optical and structural characterizations of the GENTs (QSY21 as the model Raman tag) using UV-vis absorption spectroscopy, NTA, and TEM. The longitudinal LSPR band of the GENTs prepared with 2 mM HAuCl_4_ was blue-shifted to 628 nm compared to that of the seeding AuNRs. The nanoparticles were uniform in their sizes, with a HD of 71.5 ± 0.8 nm, which increased by approximately 24 nm compared to that of the AuNRs. TEM imaging revealed that the resulting GENTs retained the rod-like morphology of the seeding AuNRs. The GENRs exhibited an average AR of 1.9 ± 0.3, with an average length of 61.6 ± 6.8 nm and width of 33.0 ± 3.7 nm. Compared to the original SERS-AuNR seeds, the GENRs showed an estimated shell growth of ~3.9 nm along the longitudinal axis and 6.5 nm along the transverse axis. These results suggest that the Au shell exhibited preferential deposition along the short axis of the seeding SERS-AuNRs. Optical and structural characterizations revealed pronounced changes in the resulting GENRs with increasing HAuCl_4_ concentration (Table 1). At 4 mM HAuCl_4_, the GENRs exhibited a further blue shift in LSPR to 599 nm and a slight increase in HD to 73.3 ± 0.3 nm. TEM analysis showed an AR of 1.7 ± 0.2, with dimensions of 64.8 ± 6.8 nm in length and 37.2 ± 3.4 nm in width. These values correspond to Au shell thicknesses of approximately 5.5 nm along the longitudinal axis and 8.6 nm along the transverse axis. Increasing the HAuCl_4_ concentration to 8 mM induced a more pronounced LSPR blue shift, with the longitudinal and transverse peaks merging into a single band at 548 nm, accompanied by an increase in HD to 86.2 ± 1.2 nm. The particles measured 67.1 ± 7.3 nm in length and 49.3 ± 5.5 nm in width, yielding an AR of 1.3 ± 0.2. These dimensions indicate shell thicknesses of approximately 6.6 nm along the longitudinal axis and 14.7 nm along the transverse axis. These findings confirm a preferential growth of the Au shell along the short axis of the seeding SERS-AuNRs.

The observed systematic trends in LSPR, particle size, and morphology of the GENRs with varying Au precursor concentrations, relative to the seeding SERS-AuNRs, indicate the formation of a Au shell around the AuNR core. It is noted that a distinct gap was not clearly visible in the TEM images, likely due to the dense packing of the CTAB bilayer and Raman reporters within the nanogap and the identical composition of the core and shell (both made of Au) which limits contrast under standard TEM imaging. In previous studies, the rod-in-shell GERT structure could be visualized by TEM when silver (Ag) was used as the shell material, whereas the nanogap created by the Raman reporter molecule remained invisible [60]. Plasmonic coupling between the core and shell rod structures generates a second LSPR band above 1000 nm [56], which is not detectable with our UV–Vis spectrometer.

To further confirm that our GENRs are indeed core–shell structures templated on AuNR seeds, rather than products of spontaneous nucleation from the Au growth solution, we performed a control experiment in the absence of SERS-AuNR seeds. Figure 2m presents the unnormalized absorption spectra of GENRs synthesized using varying concentrations of HAuCl_4_, alongside the control sample without adding the SERS-AuNR seeds. The results show that the longitudinal LSPR peaks varied in both intensity and position depending on the HAuCl_4_ concentration, with higher precursor concentrations producing GENRs with stronger absorption intensities. In contrast, no nanoparticle formation was observed when SERS-AuNR seeds were omitted from the growth solution. These findings confirm that the formation of GENRs is seed-mediated, with Au shells grown on the AuNR cores, rather than arising from spontaneous nucleation.

Further evidence supporting the gap-enhanced structure of the resulting nanoparticles is their superior stability during centrifugation and washing, as compared to SERS-AuNRs (Figure 2n). Although the SERS-AuNRs were protected by PEGylation, their Raman signal intensity decreased by over 50% after three rounds of centrifugation (8160× *g*, 10 min) and washing with water. In contrast, the GENRs exhibited only a ~14% reduction in SERS signal under the same conditions. These results strongly suggest that the Raman reporter in GENRs was securely embedded within the nanogap between the AuNR core and the outer Au shell, providing both enhanced protection and robust signal retention. Figure 2o compares the SERS activities of the Raman nanotags (QSY21 as the Raman reporter) between the traditional SERS-AuNRs and GENRs (4 mM HAuCl_4_ in the growth solution as the model) at the same nanoparticle concentration (0.5 nM). The GENRs exhibited approximately threefold higher SERS signals than the SERS-AuNRs. This suggests that the Raman reporter in GENRs was indeed embedded within the nanogap between the AuNR core and the external Au shell, thereby conferring the characteristic gap-enhanced SERS properties.

The formation of core–shell structures is an interplay of thermodynamic and kinetic parameters, which are strongly influenced by the temperature, chemical composition and concentrations in the growth solution, and the surface chemistry of the seeding nanoparticles [69]. In addition, the density of Raman reporters also plays an important role in determining the final morphology of the shell. As investigated by Khlebtsov et al., uniform rod-in-shell GERTs could only be achieved with low surface density of Raman molecules [60]. By finely controlling the synthetic parameters, we have made rod-in-shell GENTs using SERS-AuNRs as the seeds and Au as the shell. The Au shell preferentially grew along the longitudinal faces of the seeding AuNRs, likely due to the electrostatic attraction of negatively charged AuCl_4_^−^ ions to the positively charged CTAB capping agents (with Raman reporter embedded in the CTAB bilayer) on these surfaces, thereby promoting anisotropic gold deposition.

### 3.3. Synthesis and Characterization of Multicolor GENRs

**Figure 3:**
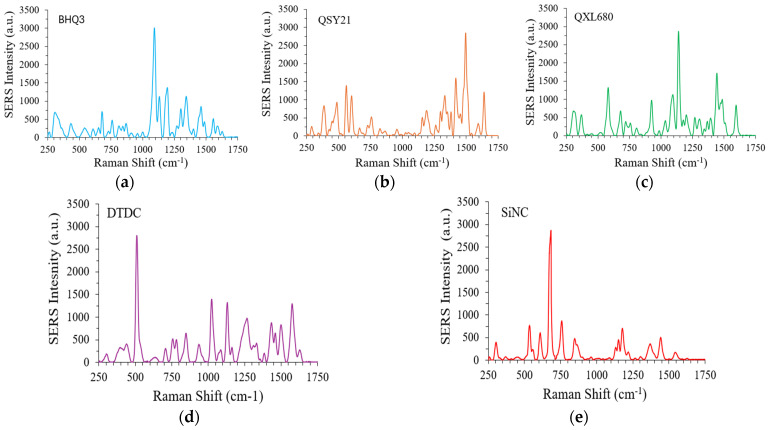

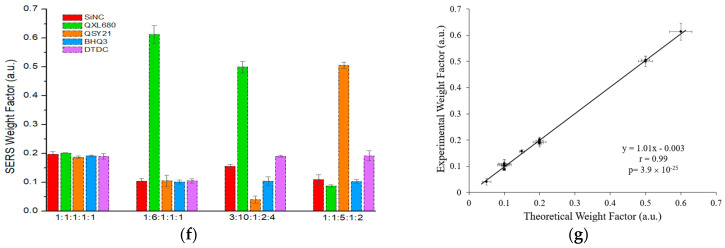
(**a**–**e**) SERS spectra of five-color GENRs (0.1 nM) encoded with BHQ3 (**a**), QSY21 (**b**), QXL60 (**c**), DTDC (**d**), and SiNC (**e**) displaying distinct Raman signatures. (**f**) Comparison of the experimentally measured and theoretical SERS weight factors for the five-color GENRs in mixed solutions. (**g**) Correlation of the data presented in (**f**). r is the Pearson’s correlation coefficient and *p* is the *p*-value for the slope indicating the significance of the linear correlation between *y* and *x*.

To report five integrin markers, five Raman reporters with distinct SERS spectra were used, BHQ3, QSY21, QXL680, DTDC, and SiNC. The SERS spectra of the GENRs (0.1 nM concentration) using these Raman reporters are shown as Figure 3a–f. The integrin α3, β1, β3, β4, and β5 was reported by SiNC, BHQ3, QXL680, QSY21, and DTDC, respectively. To facilitate comparative studies, the ratio of Raman reporter to AuNRs was adjusted to make SERS-AuNRs with similar SERS intensity at their representative peaks, BHQ3 at 1094 cm^−1^, QSY21 at 1496 cm^−1^, QXL680 at 1140 cm^−1^, DTDC at 510 cm^−1^, and SiNC at 684 cm^−1^ (the strongest peak of each reporter).

To examine the accuracy of GENRs for multiplexed detection, we mixed the five color GENRs in various ratios, measured total SERS signals, and deconvolved the signals for each color using CLS. The results show that the experimentally measured contribution of each color tag matched well with their theoretical ones (Figure 3f,g, Table S1). For example, in the SiNC/QXL680/QSY21/BHQ3/DTDC = 1:6:1:1:1 sample, the theoretical contribution of SiNC, QXL680, QSY21, BHQ3, and DTDC was 0.10, 0.60, 0.10, 0.10, and 0.10, respectively. The experimentally measured contribution of these tags was 0.10 for SiNC, 0.61 for QXL680, 0.10 for QSY21, 0.10 for BHQ3, and 0.10 for DTDC, which well matched the theoretical values. Overall, the experimental results gave an excellent correlation with the therapeutic ones, with a Pearson correlation coefficient (r) of 0.99 with *p* < 0.00010.

### 3.4. Preparation and Characterization of Target-Specific GENRs

Monoclonal mouse antibodies were used as the ligand to recognize specific integrin receptors. The antibodies were first functionalized with thiol groups via the bifunctional linker NHS-PEG-SH (MW = 1000) and purified with a centrifugal filter (MWCO = 10,000 Da). Then, the thiolated antibody was conjugated to GENRs (antibody-to-GENR molar ratio: 5000) through incubation at 4 °C overnight. The surface of the antibody-conjugated GENRs was saturated with mPEG-SH (MW = 5000) to minimize nonspecific binding. Our previous studies showed that this procedure produces antibody surface density of 8.0 × 10^−4^ antibodies/nm^2^, which corresponds to 13 antibodies per GENR [34].

**Figure 4:**
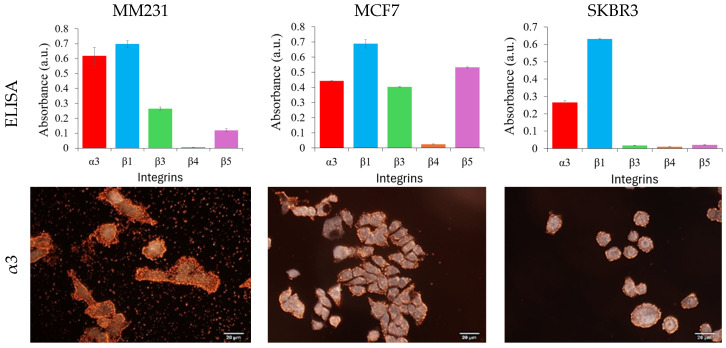

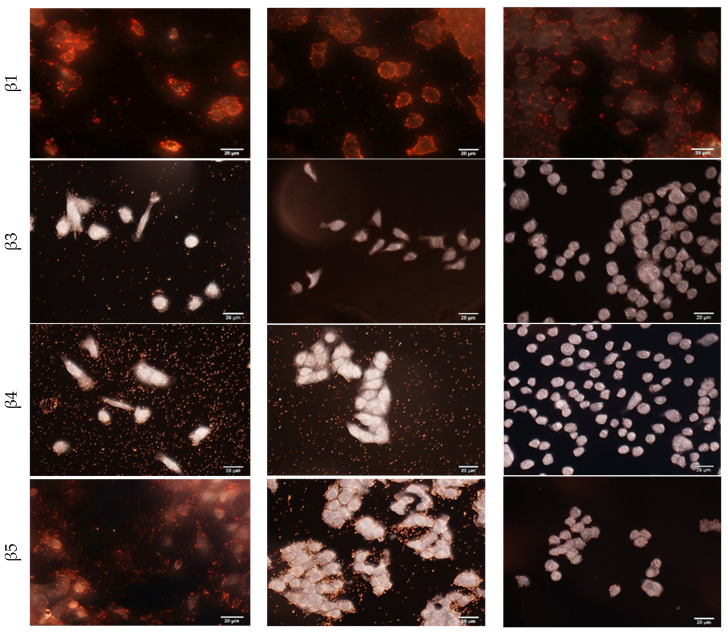
Characterizations of integrin expression on breast cancer cells with ELISA (**row 1**) and cellular binding of target-specific GENRs (**rows 2**–**6**). ELISA data were corrected with control using isotype IgG.

The specificity of the antibody-conjugated GENRs was examined by their cellular binding to three breast cancer cell lines, MM231, MCF7, and SKBR3 cells. These cell lines represent triple negative, hormone positive, and HER2-positive breast cancer, respectively. The integrin expressions on these cell lines were first characterized with cellular ELISA, a gold-standard method to measure the expression level of cellular surface markers at the bulk level. The results show that MM231 cells exhibit high expression of integrins α3 and β1, moderate expression of β5, and no expression of integrin β3 (Figure 4, first row and Figure S4). MCF7 cells display high levels of integrin β1, followed by β5.

We also observed α3 and β3, but no detectable level of β4. SKBR3 cells exhibit the highest expression of integrin β1, followed by α3, while showing no detectable expression of β3, β4, or β5. Clearly, these cells are distinct in terms of the expression profile of the five integrins, giving an opportunity to distinguish them from a cocktail by the expression of the composite integrin markers.

Using the MM231, MCF7 and SKBR3 cells, specific cellular binding of each antibody-conjugated GENRs as well as IgG-conjugated GENRs, was examined with dark field imaging (Figure 4, rows 2–6, Figure S5). The IgG-conjugated control nanoparticles (QSY21 as the model Raman reporter) exhibited no binding to any of the tested cell lines. Further characterization with Raman spectroscopy showed that the Raman spectra obtained from cells of each cell line (averaged Raman spectrum from 10 different cells) treated with IgG-conjugated control nanoparticles closely resembled those of untreated cells. This spectral similarity indicates a lack of binding between the IgG control nanoparticles and the cell surface (Figure S6). The cellular binding of the antibody-conjugated GENRs agreed with the expression profile of each integrin on all three cell lines measured by ELISA. For example, integrin α_3_ has the highest expression level on MM231 cells among the three cell lines. SKBR3 cells are positive for integrins α3 and β1 but negative for β3, β4, and β5. The individual particles in the background of the dark field images for MM231 cells targeting integrin α3, MM231 cells targeting β4 and MCF7 targeting β4 were due to nonspecific binding of the targeting nanoparticles to 8-well chamber slides. These nonspecific interactions do not affect the Raman signals from single cells, as unbound nanoparticles are removed from the cell–nanoparticle suspension after the binding step and prior to Raman measurement.

### 3.5. Multiplexed Integrin Detection with Five-Color GENRs

To examine the ability of the multicolor GENRs to detect multiple integrins simultaneously, we incubated each of MM231, MCF7, and SKBR3 cells suspended in DPBS with a cocktail of five-color nanotags for 30 min at 37 °C with constant mixing. Since the IgG control nanoparticles did not bind to cells (Figures S5 and S6), their inclusion in the multiplexed color detection was deemed unnecessary. It is worth noting that the signal readout in both SERS method and ELISA relies on antibody–antigen interactions, and variations in antibody affinity or epitope accessibility may lead to differences in the apparent expression levels of integrins. To minimize such variability, we used the same antibody for both methods. Furthermore, the antibody was obtained from a reputable commercial source (BioLegend, San Diego, CA, USA) to ensure high specificity, consistent quality, and reproducible binding affinity across all experiments. The labeled cells were separated from free nanotags by low-speed centrifugation. SERS signals from individual cells were collected and deconvolved using CLS to obtain the SERS weight factor of each nanotag using the individual reference spectrum of the five nanotag solutions. This deconvolution is based on the fact that total Raman signals are linearly proportional to the signals of individual components. The SERS weight factor represents the ratio of the SERS signal intensity for a specific nanotag on a cell to its reference spectrum and it can be of varying value depending on the level of the targeted integrin on the cells.

**Figure 5:**
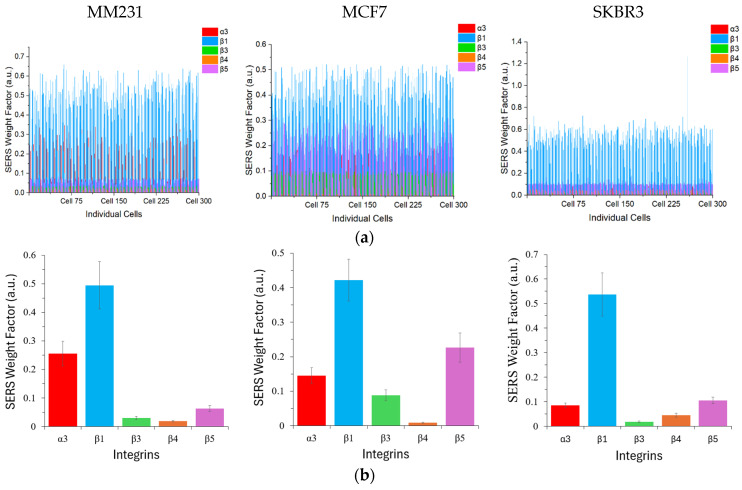
Five-plex integrin detection of breast cancer cells by GENRs. (**a**) SERS weight factor from single cells (n = 300) for each cell line. (**b**) Mean SERS weight factor from (**a**). The SERS weight factors varied according to the cell type, integrin type, and among the individual cells within the same cell line. Integrin β1 exhibited the highest expression across all three cell lines whereas β4 showed no to weak expression in all cell lines.

Figure 5a shows the SERS weight factor of each color GENRs on individual cells. The SERS weight factors varied significantly, from near-zero to 0.66, depending on the cell type, integrin type, and even among cells of the same line due to the heterogeneous surface protein expression at the single-cell level. To statistically compare the SERS weight factors across integrins and cell lines, we calculated the mean values and standard deviation of each marker for each cell line (Figure 5b).

As described above, ELISA and SERS quantify protein expression based on fundamentally different principles. In the ELISA, the abundance of a targeted surface protein is determined by the TMB absorbance intensity, whereas in the SERS method, it is represented by the ratio of the SERS nanotag signal on a cell to the reference SERS spectrum (the SERS weight factor). Therefore, the absolute values obtained for a given protein from these two methods are not expected to be identical, even though both approaches provide quantitative measurements of the target protein’s expression level. To examine the accuracy of the SERS detection, we compared the results using the SERS mean values with those from cellular ELISA shown previously in Figure 3 for each cell line. As shown in Figure S7, the SERS method correlated linearly with the ELISA method, with a Pearson’s r greater than 0.9 for all three cell lines. Using the three representative cell lines of three breast cancer subtypes, we have demonstrated that five distinct integrins can be simultaneously detected on cancer cells by the five-color GENRs.

### 3.6. Multiplexed Detection of Spiked CTCs in PBMCs

**Figure 6:**
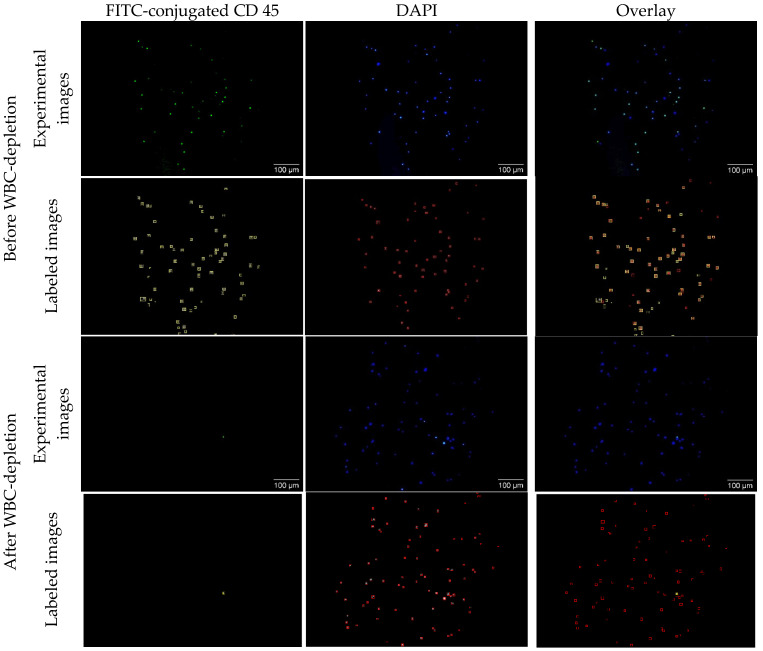
Fluorescence characterization of the WBC-depletion efficiency with CD45-magnetic nanobeads. CD45-FITC was used to identify WBCs and DAPI for both WBCs and cancer cells. First and second rows: MM231 spiked PBMCs before WBC-depletion. Third and fourth rows: MM231 spiked PBMCs after WBC-depletion. First and Third rows: Experimental fluorescence images. Green squares represent CD45-positive cells while red squares represent DAPI-positive cells. Second and fourth rows: Labeled images. Yellow squares represent CD45-positive cells while red squares represent DAPI-positive cells.

Typical CTC concentrations range from 1–10 CTCs per 10 mL of whole blood in early-stage disease and 10–1000 CTCs per 10 mL in metastatic stages. The PBMC fraction provides ≥10-fold CTC enrichment compared to whole blood. In this study, we intentionally used a high number of spiked cancer cells (~50,000 cells) in PBMC suspensions. This elevated concentration does not reflect physiological levels in patient samples but was selected to facilitate technology development, optimization, and validation under controlled conditions. While CTCs are enriched in the PBMCs after density centrifugation of whole blood, WBCs are also enriched in PBMCs due to their similar sizes and densities. To remove the WBCs, we used magnetic beads (MBs) coated with CD45 antibodies to separate WBCs from spiked cancer cells in PBMCs. To evaluate the efficiency of WBC depletion, the WBC-depleted PBMC samples were stained with both DAPI and FITC-conjugated CD45 antibodies. While DAPI (blue) detects both cancer cells and WBCs, FITC-conjugated CD45 (green) only detects WBCs. To calculate the depletion efficiency, cells in each image were counted with auto-SEDIA, a fast method we recently developed for dual imaging analysis [64]. Figure 6 shows representative fluorescence images and the corresponding machine learning-generated images for a non-depleted and depleted sample. Before depletion, the algorithm classified 50 of the 65 (76%) observed cells as WBCs. After WBC-depletion, the machine learning algorithm determined that 1 of the 88 (0.01%) observed cells was a WBC, giving the WBC depletion efficiency of 99.99%. With the analysis of multiple pairs of images in duplicated experiments, we determined the WBC-depletion efficiency to be 99.96%, suggesting extremely high efficiency of magnetic separation using the CD45-conjugated magnetic nanobeads.

**Figure 7:**
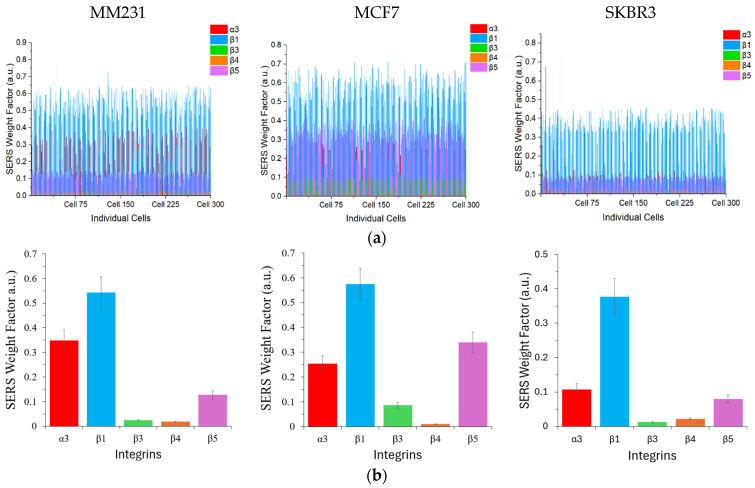
Five-plex integrin detection of spiked CTCs in PBMCs by GENRs. (**a**) SERS weight factor from single cells (n = 300) for each cell line. (**b**) Mean SERS weight factor from (**a**). The SERS weight factors varied according to the cell type, integrin type, and among the individual cells within the same cell line. Integrin β1 exhibited the highest expression across all three cell lines whereas β4 showed no to weak expression in all cell lines.

After WBC depletion and negative staining with DAPI and FITC-conjugated CD45 antibodies, the cancer cells were placed on a quartz slide and covered with a coverslip for Raman analysis using a 40× objective to allow for single-cell observation. Only the DAPI-positive and FITC-negative cells were subjected to SERS measurement. Similarly to the studies of pure MM231 cells, single-cell SERS signals were collected and the SERS weight factor for each color GENRs was obtained with the CLS-based deconvolution (Figure 7a). The mean weight factor with standard deviation across 300 cells was calculated (Figure 7b). Similarly to the results when the cells were suspended in DPBS (Figure 4), the SERS weight factors exhibited considerable variability from zero to 0.79. This variation can be attributed primarily to differences in cell type, integrin type, and inherent heterogeneity among individual cells within the same cell line. Importantly, the robustness of the SERS method was validated by its strong linear correlation with traditional ELISA measurements of integrin expression, achieving Pearson’s r greater than 0.9 across all three breast cancer cell lines tested (Figure S8). This high correlation underscores the quantitative reliability and accuracy of the SERS approach as a complementary analytical tool to ELISA.

**Figure 8:**
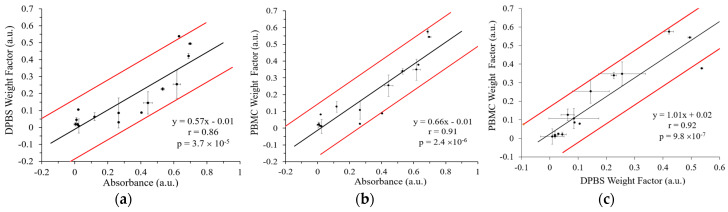
Correlation between the five-plex SERS methods and ELISA. (**a**) SERS detects cells suspended in DPBS versus ELISA. (**b**) SERS method of cells suspended in PBMCs versus ELISA. (**c**) DPBS versus PBMC for the SERS method. The black line is the linear fit and the red lines represent the uncertainty bounds. The SERS methods for multiplexed detection of cancer cells suspended in DPBS and in PBMCs demonstrated a strong correlation to ELISA (Pearson’s r > 0.85) and mutual correlation (Pearson’s r = 0.92). r is the Pearson’s correlation coefficient and *p* is the *p*-value for the slope indicating the significance of the linear correlation between *y* and *x*.

Figure 8 shows the correlations of the five-plex SERS methods with ELISA and with each other by combining the data from the five integrins of all three cell lines. The Peason’s r for the SERS method to detect cancer cells in DPBS and ELISA was 0.86 and that for the SERS method to detect spiked cancer cells in PBMCs and ELISA was 0.91. This high correlation again demonstrates the robustness of the GENRs for multiplexed SERS detection. Moreover, the single-cell resolution provided by SERS enables detection of cellular subpopulations that might otherwise remain masked in ensemble ELISA measurements, thereby offering deeper insights into the complexity of tumor cell biology. Overall, these findings reinforce the utility of the multiplexed SERS-based approach with five-color GENRs for precise, single-cell-level characterization of heterogeneous biomarker expression in cancer research. In addition, comparison of the SERS methods for multiplexed detection of cancer cells suspended in DPBS and in PBMCs demonstrated a strong mutual correlation (Pearson’s r = 0.92), further confirming the reliability and consistency of SERS-based quantification. Collectively, these findings reinforce the utility of the multiplexed SERS-based approach for precise, single-cell-level characterization of heterogeneous biomarker expression in cancer research.

Overall, the representative TNBC (MM231), hormone-positive (MCF7), and HER2-positive (SKBR3) cells exhibit distinct integrin profiles, providing a great opportunity to detect them based on these expression patterns. Among the five integrins, integrin β1 was highly expressed on all three cell lines. Integrin α3 also showed strong expression on these cells. Integrin β5 showed markedly higher expression on the hormone-positive breast cancer cells than the other two subtypes. The strong expression of these integrins on multiple breast cancer cell lines is not surprising as they have been shown to associate with increased metastatic potential through the promotion of cell adhesion, migration, and signal transduction [70,71,72]. Integrin β1, particularly, plays a key role in ECM interaction and can serve as an activation mechanism for estrogen response enhancement [73,74]. It is not only important for the attachment of cancer cells to the extracellular matrix but also for the signaling pathways that regulate cancer cell survival, proliferation, and metastasis [75,76,77,78,79].

### 3.7. Detection and Classification of Breast Cancer Subtypes by Machine Learning Algorithm

**Figure 9:**
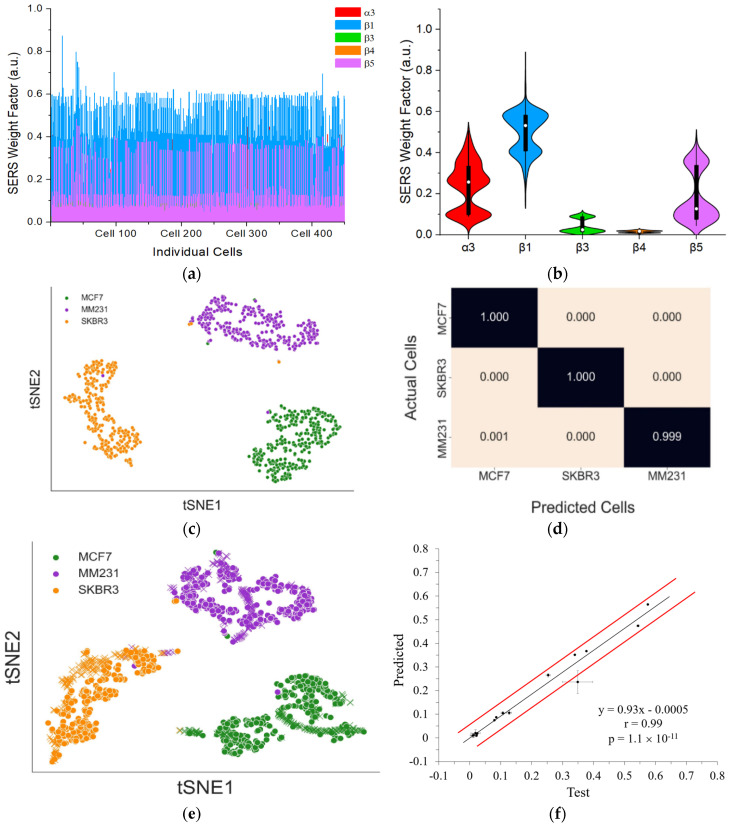
Detection and classification of breast cancer subtypes using integrin profiles and machine learning-based data analysis. (**a**) SERS weight factor for each marker from 450 SKBR3, MM231, and MCF7 mixed cells. (**b)** Violin plot showing the distribution of each cancer marker expressions on single cells. (**c**) tSNE visualization of the labeled training data. (**d**) Confusion matrix from the training data with the actual cells along the *y*-axis and the predicted cells along the *x*-axis. Values are presented as fractions. (**e**) tSNE visualization of the cancer cells in the training set along with the predicted cells in the test set. Crosses represent predicted cell types and dots represent cells in the training set. (**f**) Correlation plot comparing the mean SERS values of five markers for the cells in the training set and the mean predicted SERS values of the markers of predicted cells. The black line is the linear fit and the red lines represent the uncertainty bounds. r is the Pearson’s correlation coefficient and *p* is the *p*-value for the slope indicating the significance of the linear correlation between *y* and *x*.

To test the feasibility of using integrin profiles to predict and classify breast cancer subtypes, we added MM231, MCF7, and SKBR3 cells (each 5 × 10^4^ cells) into PBMCs that was extracted from a healthy donor. Following the same sample preparation procedure as detecting pure cancer cells of each cell line, SERS signals from 450 individual cells were collected and deconvolved to obtain the SERS weight factor from each of the five-color GENRs (Figure 9a). Mean values with standard deviation from these cells were calculated, which showed the expression profile of each integrin on the mixed cancer cells (Figure 9b).

To detect and differentiate the three subtypes of cancer cells in the mixture, a random forest model was developed using the integrin profile of pure cancer cells of the three cell lines determined with the spiked PBMC samples (data in Figure 7). Before serving training data, the deconvoluted SERS weight factors were projected onto a two-dimensional (2D) plane using t-SNE, an unsupervised learning technique (Figure 9c). This approach enabled comparison and visualization of the relationships between the different cell lines and their distinct protein expression patterns in an unbiased manner. We selected this nonlinear dimensionality reduction method because t-SNE effectively compresses high-dimensional data into a lower-dimensional representation while preserving the relative distances between data points. Due to the stochastic nature of t-SNE, the visualization results can vary across iterations. To address this, we generated five different t-SNE mappings for each perplexity value ranging from 5 to 300. The optimal clustering result was chosen based on the lowest Kullback–Leibler (KL) divergence value.

To assess the quality of clustering in the selected t-SNE plots, we applied the Davies−Bouldin Index (DBI) to determine the most suitable perplexity value. The t-SNE plot generated with a perplexity value of 45 exhibited the lowest DBI, making it the most representative visualization of the original deconvoluted data. The results demonstrated that each cancer cell line could be effectively clustered and distinguished from the others using unsupervised learning techniques. Given this success, we applied supervised learning to classify each cell line using labeled data, where the deconvoluted data for each cell line was pre-identified. This approach allowed us to predict the class of each cell in a mixed population of all three cell lines.

The labeled deconvoluted data, which was previously used to generate the t-SNE mappings, was utilized in a random forest classifier implemented using the scikit-learn package in Python. The classification model was trained as a multiclass random forest, where each cell line was assigned to a distinct class. The dataset was split into 70% training data and 30% test data to evaluate model performance. The model underwent 200 training iterations, after which its performance was assessed using average F1 scores and confusion matrices. The random forest model achieved an impressive average F1 score of 0.99, demonstrating strong classification accuracy. The F1 score is a widely used metric for evaluating the effectiveness of random forest models. This score integrates two key performance measures: precision (PRE) and recall (REC), both of which are derived from the confusion matrix (Figure 9d). The final F1 score is computed using the following formula:

$$F1=\frac{2\times PRE\times REX}{PRE+REC}$$

The model was then retrained using the entire training dataset to enhance its ability to label cells accurately before being applied to predict the class of each cell in a mixed solution of three cell lines. The trained model classified mixed cells as 148 MCF7 cells, 22 SKBR3 cells, and 280 MM231 cells from a set of 450 randomly selected cells.

To visually evaluate the performance of the random forest model, a t-SNE plot was generated. As anticipated, cells predicted to belong to the same class clustered closely together, demonstrating the effectiveness of the model in distinguishing between different cell populations, as illustrated in Figure 9e. Furthermore, a correlation analysis between the mean values of the deconvoluted predicted cell data and those from the training dataset was conducted, resulting in a Pearson’s r of 0.99 (Figure 9f). This exceptionally high correlation underscores the precision and robustness of the prediction model. These results collectively demonstrate that the random forest model reliably classifies distinct cell lines based on SERS spectral data, highlighting the potential of this approach for advanced cancer diagnostics and personalized therapeutic strategies.

## 4. Conclusions

We have demonstrated a robust, sensitive, and accurate platform for multiplexed detection and characterization of integrin expression at the single-cell level, enabling the precise classification of different breast cancer subtypes. Our approach leverages the unique optical properties and enhanced stability of GENRs coupled with the powerful multiplexing capability of the SERS technology, allowing for simultaneous detection of five distinct integrins (α3, β1, β3, β4, and β5) on breast cancer cell surfaces. By employing antibodies conjugated to GENRs, we successfully profiled integrin expression on individual cancer cells in both controlled buffer conditions (DPBS) and complex biological matrices (human PBMCs). The strong correlation between SERS-based detection and conventional cellular ELISA validates the accuracy and reliability of our platform.

Furthermore, integrating our multiplexed SERS data with advanced computational analyses, including CLS regression for signal deconvolution and a random forest ML classifier, we achieved exceptional classification accuracy (99.9%) for distinguishing three breast cancer cell lines in mixed populations. This demonstrates the platform’s potential for precise cancer subtype characterization, which is critical for personalized diagnostics and targeted therapeutic interventions. Overall, our study establishes a highly sensitive and multiplexed analytical method for integrin-based molecular profiling of cancer cells, advancing cancer diagnostics and offering significant potential for clinical applications, such as the identification and analysis of CTCs. Future developments may extend this approach to broader biomarker panels and clinical samples, further enhancing its utility in personalized medicine and cancer management.

Comparing to previous GERTs or SERS nanotag systems, this GENR-based multiplexing system exhibits the following unique advantages: (1) the GENRs produce stronger and more stable SERS signals than conventional SERS nanotags, because the Raman reporter is encapsulated within a rigid Au shell rather than being coated on the surface or embedded in a soft polymer layer; (2) the GENRs exhibit greater chemical stability than previously reported Au@Ag GERTs, because they employ an inert Au shell instead of a reactive Ag coating; and (3) our GENR-based multiplexing system combines physical multiplexing (SERS method) with computational precision (machine learning) for automated cancer cell classification and CTC profiling at the single-cell level, which have not been achieved with previous GERT or SERS nanotag systems.

While the current assay demonstrates feasibility for single-cell SERS analysis, its speed remains a limiting factor for clinical application. At present, it requires approximately 10 s to acquire the SERS spectrum from a single cell. For a typical patient sample containing 100 CTCs, spectral acquisition alone takes about 16.7 min. In addition, signal deconvolution using the SOLO + MIA software requires approximately 30–40 min to compute SERS weight factors for 100 cells. As a result, the total processing time from data acquisition to output is approximately one hour per patient. However, this workflow is amenable to automation, which could significantly improve throughput and make the assay suitable for clinical implementation.

## Figures and Tables

**Table 1:** Optical and structural characterizations of AuNRs and GENRs.

	AuNRs	GENRs		
		2 mM HAuCl_4_	4 mM HAuCl_4_	8 mM HAuCl_4_
LSPR (nm)	676	628	599	548
HD (nm)	47.0 ± 0.6	71.5 ± 0.8	73.3 ± 0.3	86.2 ± 1.2
AR	2.8 ± 0.6	1.9 ± 0.3	1.7 ± 0.2	1.3 ± 0.2
Length (nm)	53.9 ± 5.7	61.6 ± 6.8	64.8 ± 6.8	67.1 ± 7.3
Width (nm)	20.0 ± 3.5	33.0 ± 3.7	37.2 ± 3.4	49.3 ± 5.5
Average thickness along longitudinal axis (nm)	-	3.9	5.5	6.6
Average thickness along transverse axis (nm)	-	6.5	8.6	14.7

## Data Availability

The original contributions presented in this study are included in the article. Further inquiries can be directed to the corresponding author.

## Supplementary Materials

The following supporting information can be downloaded at https://www.mdpi.com/article/10.3390/nano15221693/s1. Additional Supporting Information provides details on on SERS weight factor experimental value of the five-color ratio test; characterizations of GENRs synthesized with different concentrations of HAuCl4, AA, and seeds; integrin expression data by ELISA before IgG correction; examination of cellular binding of IgG-conjugated GENRs with dark field imaging and Raman spectroscopy; and correlation among the SERS method with cells suspended in DPBS, SERS method with cells suspended in PBMCs and ELISA for each cell line. Table S1: Experimental SERS weight factor determined from the five-color ratio test. Theoretical data were presented in parenthesis; Figure S1: Characterizations of GENRs synthesized with different concentrations of HAuCl_4_. (a) HDs measured by DLS. (b) LSPR spectra. (c) Photographic images. (d) Normalized Raman intensity using the SERS signal (the peak at 1497 cm^−1^) from the GENRs that gave highest intensity; Figure S2: Characterizations of GENRs synthesized with different concentrations of AA. (a) HDs measured by DLS. (b) LSPR spectra. (c) Photographic images. (d) Normalized Raman intensity using the SERS signal (the peak at 1497 cm^−1^) from the GENRs that gave highest intensity; Figure S3: Characterizations of GENRs synthesized with different concentrations of SERS-AuNR seeds. (a) HDs measured by DLS. (b) LSPR spectra. (c) Photographic images. (d) Normalized Raman intensity using the SERS signal (the peak at 1497 cm^−1^) from the GENRs that gave highest intensity; Figure S4: Characterization of integrin expression on breast cancer cells with ELISA. Signals were presented before IgG correction; Figure S5: Examination of cellular binding of IgG-conjugated GENRs with dark field imaging. (a) Cells treated with IgG-conjugated GENRs. (b) Cells only; Figure S6: Examination of cellular binding of IgG-conjugated GENRs with Raman spectroscopy. Signals from the cancer cell only were used for comparison. Each spectrum was an average from 10 cells. QSY21 was used as the Raman reporter; Figure S7: Correlation between the SERS method (with cells suspended in DBPS) and ELISA. r is the Pearson’s correlation coefficient and *p* is the *p*-value for the slope indicating the significance of the linear correlation between y and x; Figure S8: Correlation between the SERS method (with cells suspended in buffy coat) and ELISA. r is the Pearson’s correlation coefficient and *p* is the *p*-value for the slope indicating the significance of the linear correlation between y and x.
