# Supplementary material for: Multiplexed Integrin Detection and Cancer Cell Classification Using Multicolor Gap-Enhanced Gold Nanorods and Machine Learning Algorithm
Source: Nanomaterials (Basel). 2025 Nov 8;15(22):1693. doi: 10.3390/nano15221693 (PMC12655822; doi:10.3390/nano15221693)
Supplement: Supplementary file 1 [file nanomaterials-15-01693-s001.zip › nanomaterials-3952310-supplementary.pdf]

# Multiplexed Integrin Detection and Cancer Cell Classification Using Multicolor Gap-Enhanced Gold Nanorods and Machine Learning Algorithm

**Suprava Shah[1], Reed Youngerman[1], Alberto Luis Rodriguez-Nieves[1], Mitchell Lee Taylor[1], William Rodney Bantom III[1], David Thompson[2], Jingyi Chen[2], Yongmei Wang[1], and Xiaohua Huang[1,*]**

[1]Department of Chemistry, The University of Memphis, Memphis, TN 38152, United States
[2]Department of Chemistry and Biochemistry, The University of Arkansas, Fayetteville, AR 72701, United States

KEYWORDS. Integrin, multiplexed detection, breast cancer, SERS, gap-enhanced gold nanorod, machine learning

**Table S1.** Experimental SERS weight factor determined from the five-color ratio test. Theoretical data were presented in parenthesis.

| Ratios | SiNC | QXL680 | QSY21 | BHQ3 | DTDC |
|---|---|---|---|---|---|
| 1:1:1:1:1 | 0.20 (0.20) | 0.20 (0.20) | 0.19 (0.20) | 0.19 (0.20) | 0.19 (0.20) |
| 1:6:1:1:1 | 0.10 (0.10) | 0.61 (0.60) | 0.11 (0.10) | 0.10 (0.10) | 0.10 (0.10) |
| 3:10:1:2:4 | 0.16 (0.15) | 0.50 (0.50) | 0.041 (0.050) | 0.10 (0.10) | 0.19 (0.20) |
| 1:1:5:1:2 | 0.11 (0.10) | 0.088 (0.10) | 0.51 (0.50) | 0.10 (0.10) | 0.19 (0.20) |

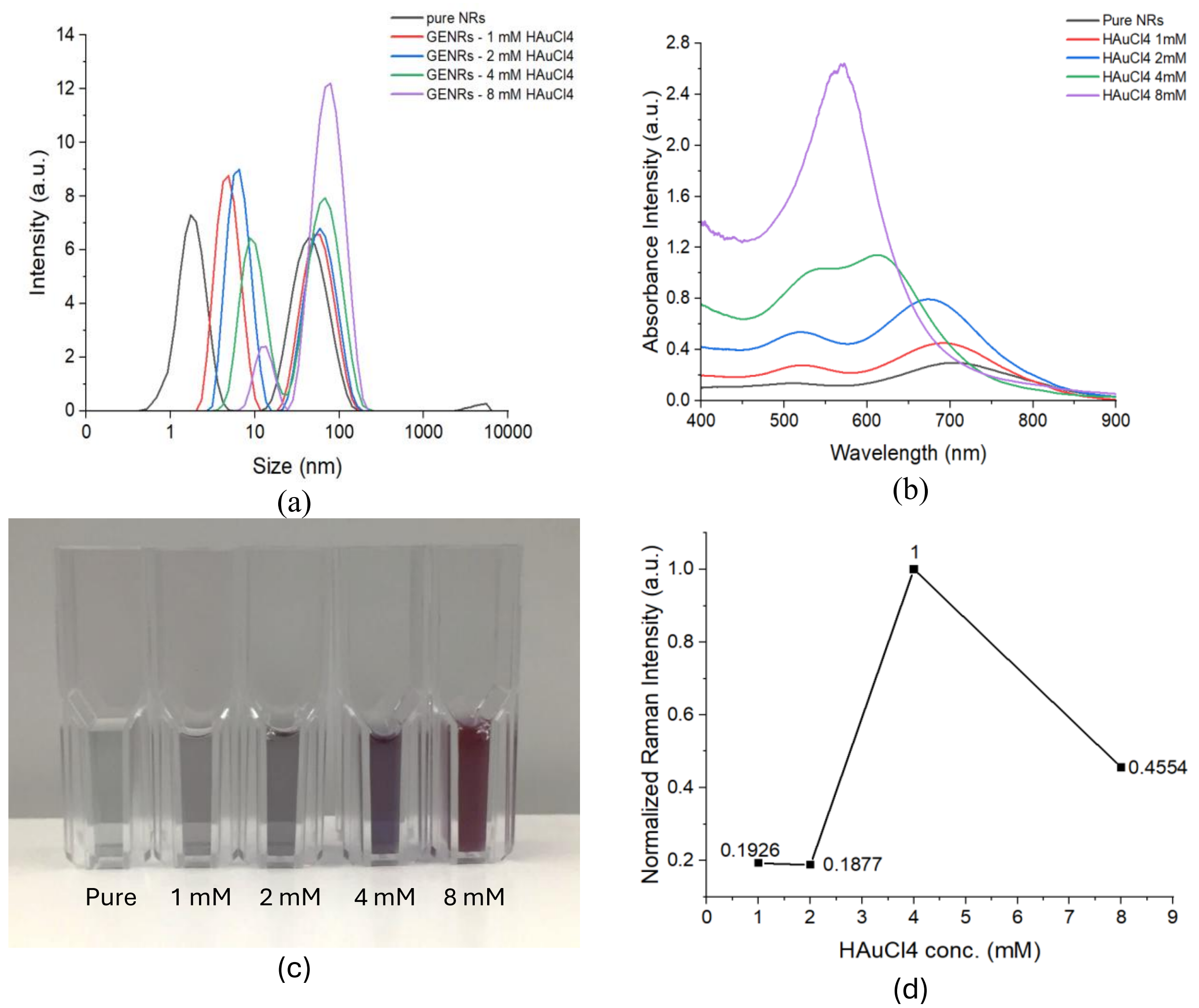


**Figure S1.** Characterizations of GENRs synthesized with different concentrations of $HAuCl_4$. (**a**) HDs measured by DLS. (**b**) LSPR spectra. (**c**) Photographic images. (**d**) Normalized Raman intensity using the SERS signal (the peak at 1497 $cm^{-1}$) from the GENRs that gave highest intensity.

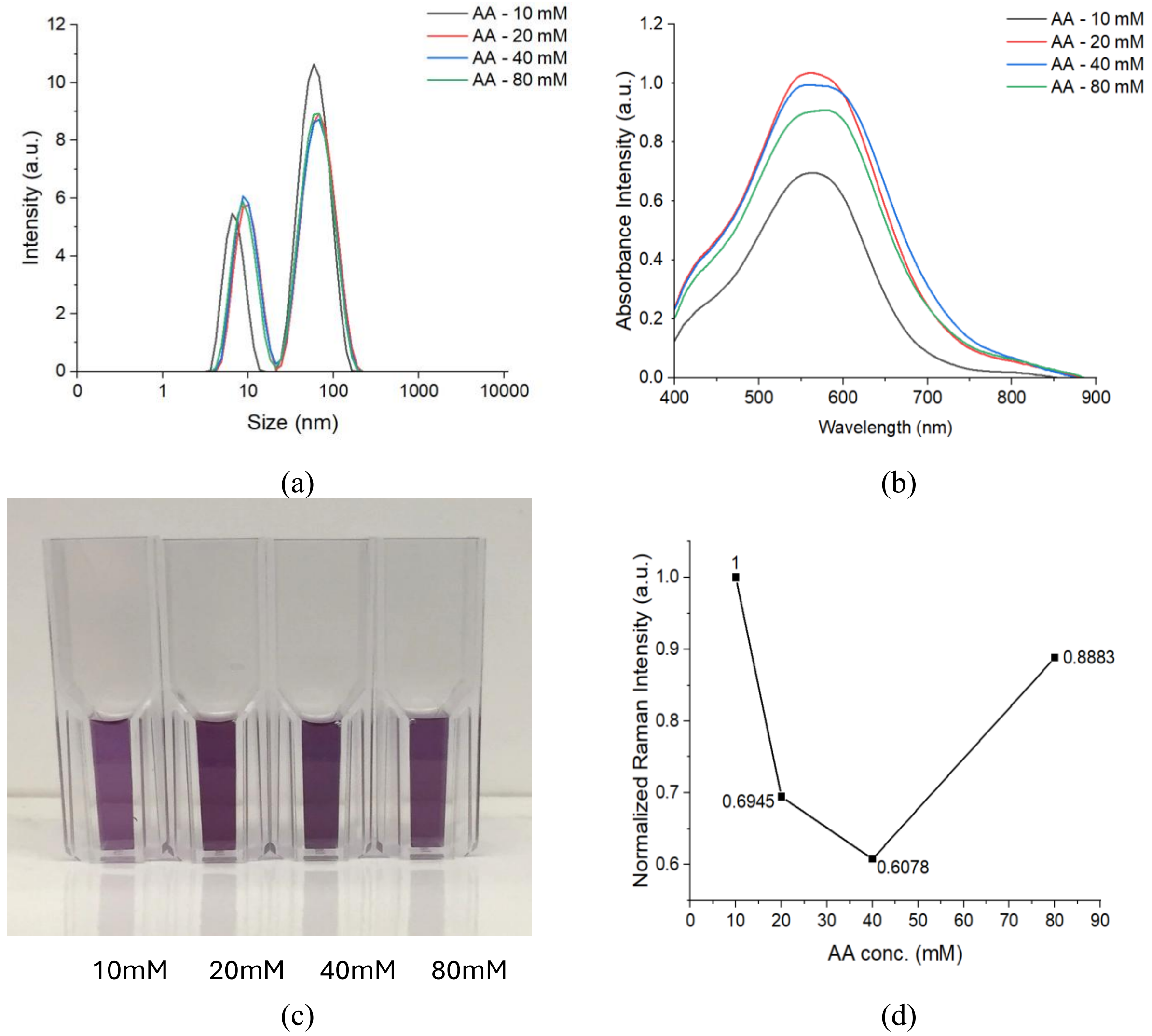


**Figure S2.** Characterizations of GENRs synthesized with different concentrations of AA. (**a**) HDs measured by DLS. (**b**) LSPR spectra. (**c**) Photographic images. (**d**) Normalized Raman intensity using the SERS signal (the peak at 1497 $cm^{-1}$) from the GENRs that gave highest intensity.

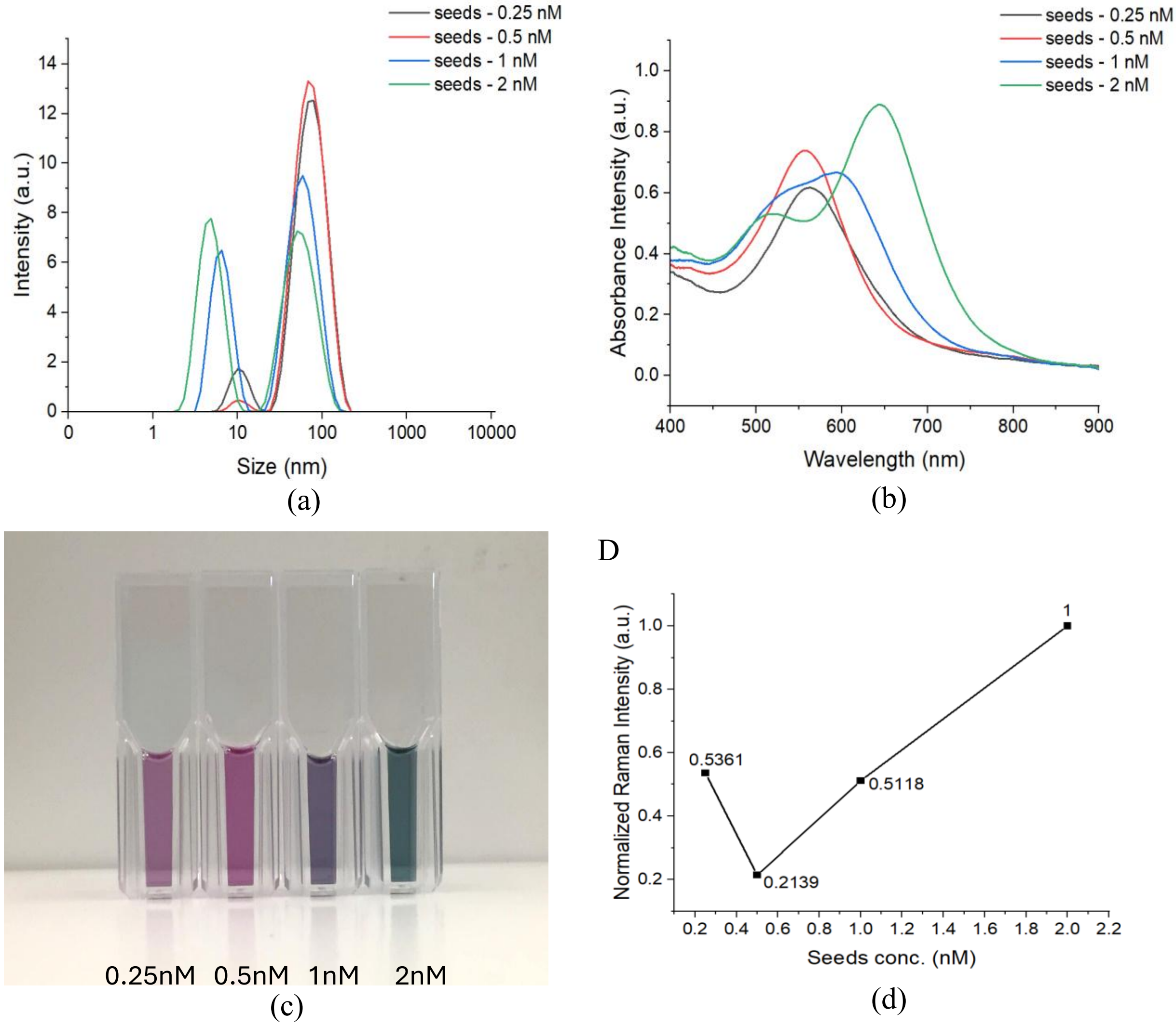


**Figure S3.** Characterizations of GENRs synthesized with different concentrations of SERS-AuNR seeds. (**a**) HDs measured by DLS. (**b**) LSPR spectra. (**c**) Photographic images. (**d**) Normalized Raman intensity using the SERS signal (the peak at 1497 $cm^{-1}$) from the GENRs that gave highest intensity.

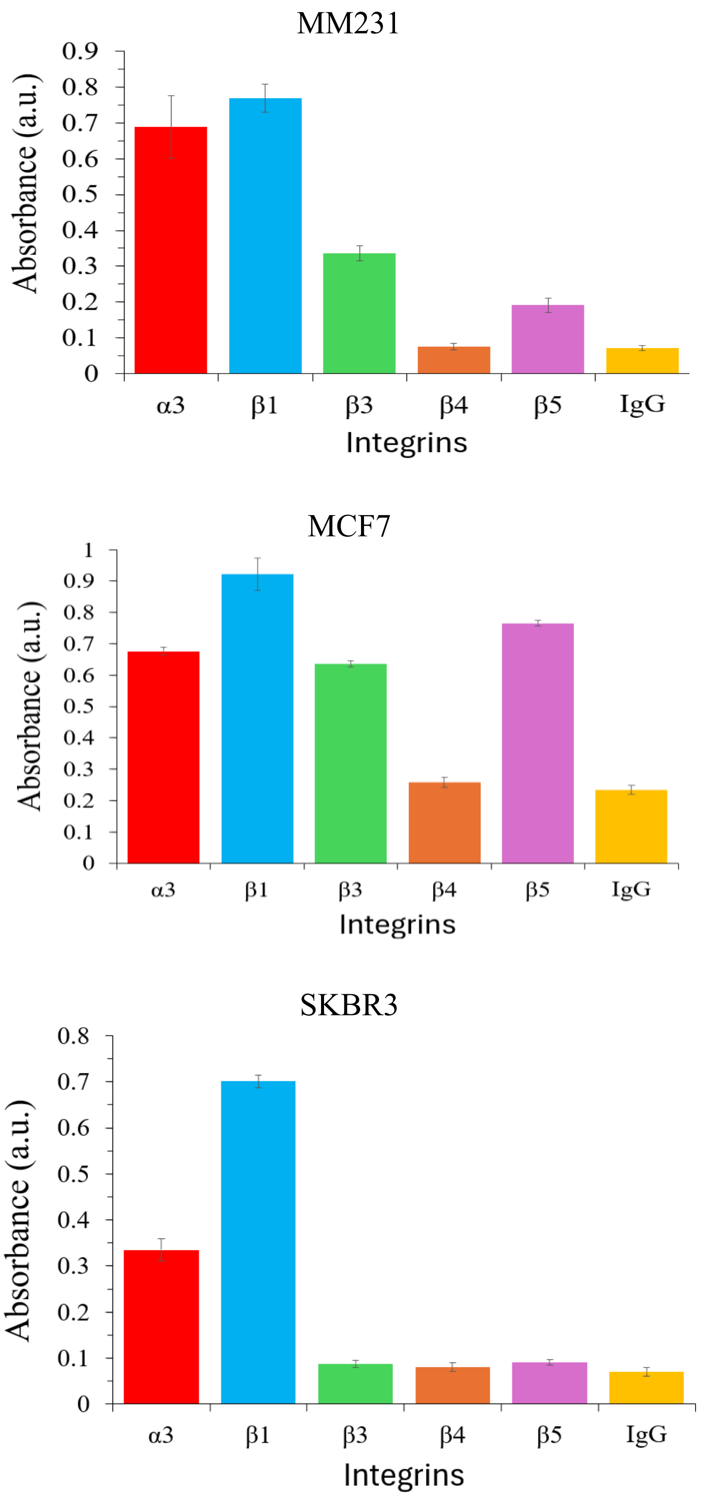


**Figure S4.** Characterization of integrin expression on breast cancer cells with ELISA. Signals were presented before IgG correction.

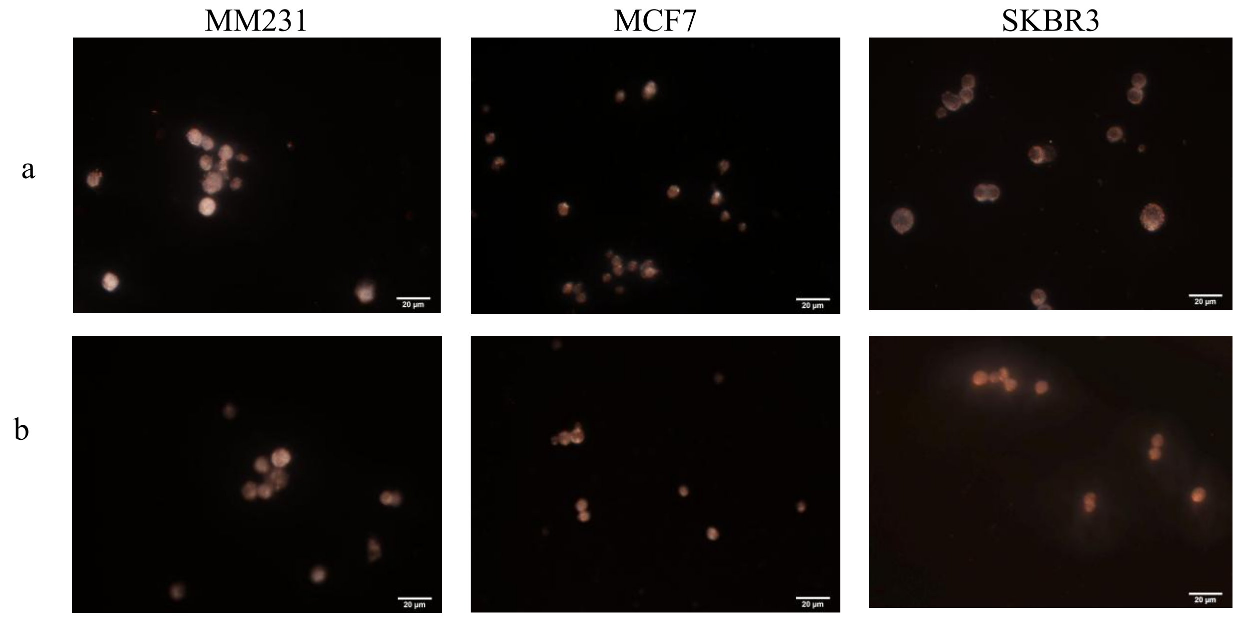


**Figure S5.** Examination of cellular binding of IgG-conjugated GENRs with dark field imaging. (a) Cells treated with IgG-conjugated GENRs. (b) Cells only.

MM231

MCF7

SKBR3

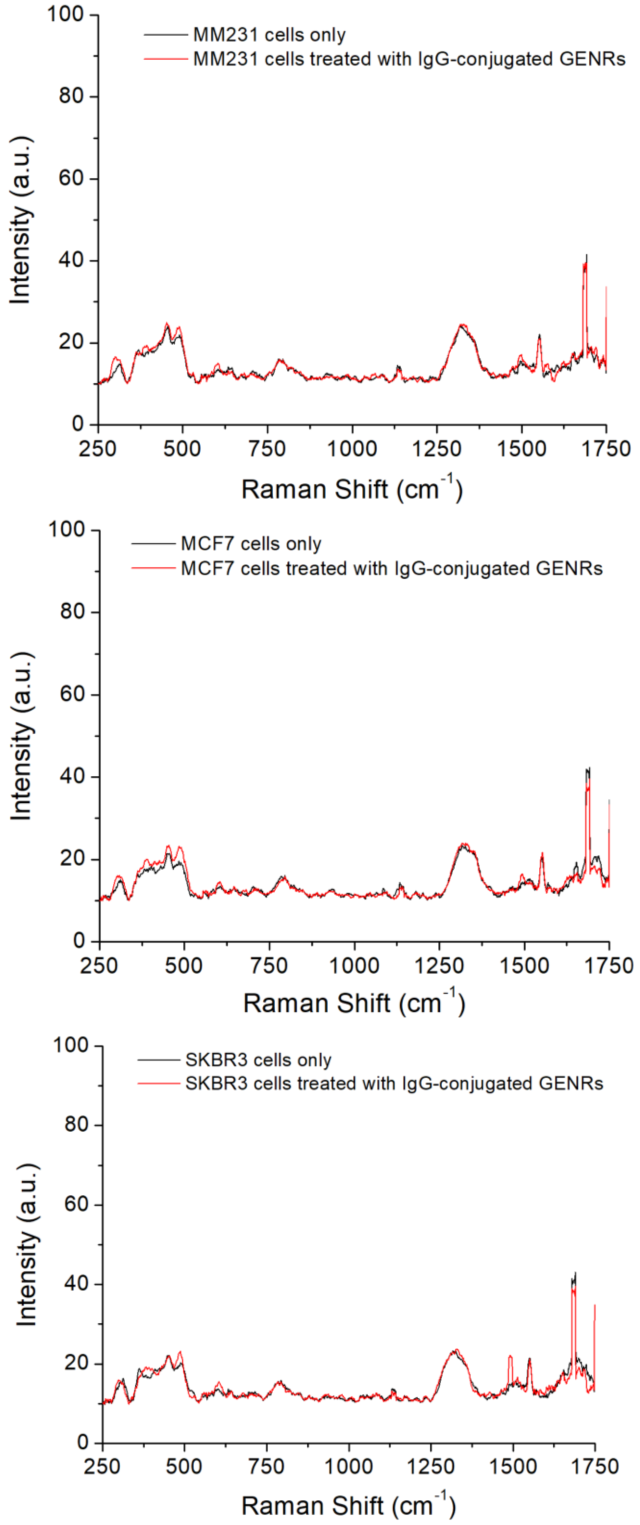


**Figure S6.** Examination of cellular binding of IgG-conjugated GENRs with Raman spectroscopy. Signals from the cancer cell only were used for comparison. Each spectrum was an average from 10 cells. QSY21 was used as the Raman reporter.

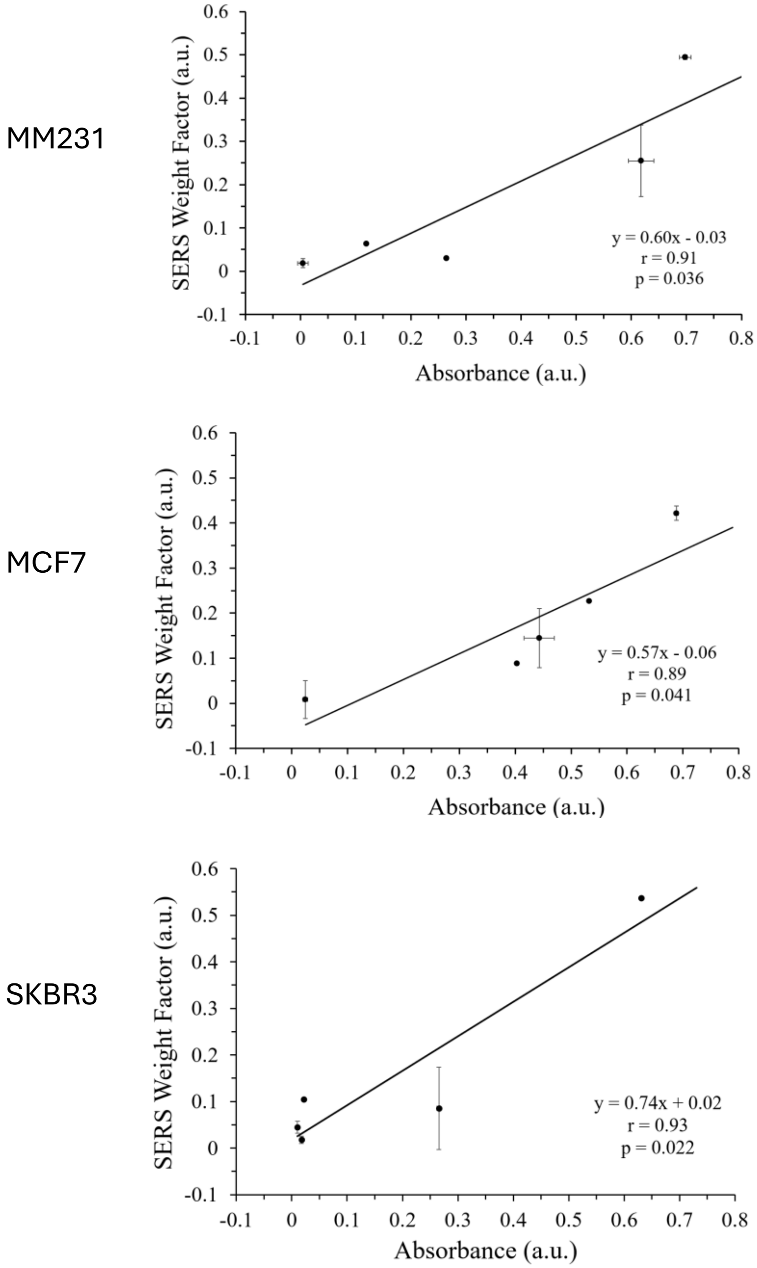


**Figure S7.** Correlation between the SERS method (with cells suspended in DBPS) and ELISA. r is the Pearson's correlation coefficient and p is the p-value for the slope indicating the significance of the linear correlation between y and x.

MM231

MCF7

SKBR3

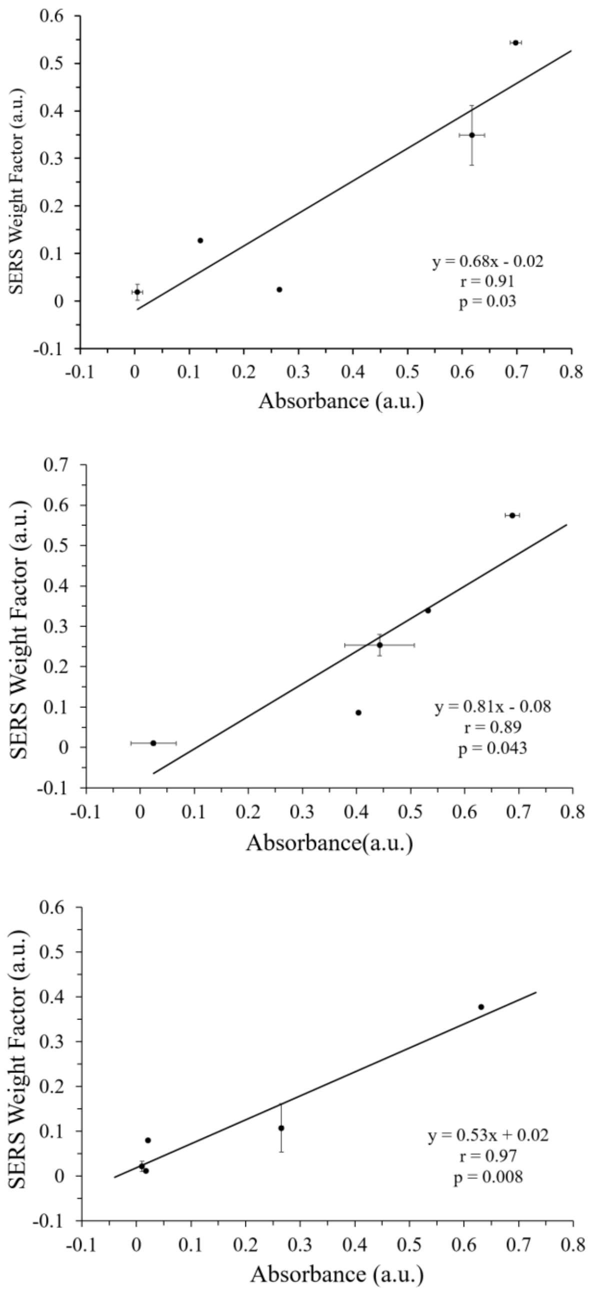


**Figure S8**. Correlation between the SERS method (with cells suspended in buffy coat) and ELISA. r is the Pearson's correlation coefficient and p is the p-value for the slope indicating the significance of the linear correlation between y and x.
